# Ruxolitinib improves hematopoietic regeneration by restoring mesenchymal stromal cell function in acute graft-versus-host disease

**DOI:** 10.1172/JCI162201

**Published:** 2023-06-20

**Authors:** Yan Lin, Quan Gu, Shihong Lu, Zengkai Pan, Zining Yang, Yapu Li, Shangda Yang, Yanling Lv, Zhaofeng Zheng, Guohuan Sun, Fanglin Gou, Chang Xu, Xiangnan Zhao, Fengjiao Wang, Chenchen Wang, Shiru Yuan, Xiaobao Xie, Yang Cao, Yue Liu, Weiying Gu, Tao Cheng, Hui Cheng, Xiaoxia Hu

**Affiliations:** 1 State Key Laboratory of Experimental Hematology, Department of Hematology, The First People’s Hospital of Changzhou, Third Affiliated Hospital of Soochow University, Changzhou, China.; 2 State Key Laboratory of Experimental Hematology, National Clinical Research Center for Blood Diseases, Haihe Laboratory of Cell Ecosystem, Institute of Hematology and Blood Diseases Hospital, Chinese Academy of Medical Sciences and Peking Union Medical College, Tianjin, China.; 3 Center for Stem Cell Medicine, Chinese Academy of Medical Sciences, Department of Stem Cell and Regenerative Medicine, Peking Union Medical College, Tianjin, China.; 4 State Key Laboratory of Medical Genomics, Shanghai Institute of Hematology, National Research Center for Translational Medicine, Shanghai Rui Jin Hospital, and; 5 Collaborative Innovation Center of Hematology, Shanghai Jiao Tong University School of Medicine, Shanghai, China.; 6 Department of Cell Biology, School of Basic Medical Sciences, Tianjin Medical University, Tianjin, China.

**Keywords:** Stem cells, Bone marrow transplantation

## Abstract

Acute graft-versus-host disease (aGVHD) is a severe complication of allogeneic hematopoietic stem cell transplantation. Hematopoietic dysfunction accompanied by severe aGVHD, which may be caused by niche impairment, is a long-standing clinical problem. However, how the bone marrow (BM) niche is damaged in aGVHD hosts is poorly defined. To comprehensively address this question, we used a haplo-MHC–matched transplantation aGVHD murine model and performed single-cell RNA-Seq of nonhematopoietic BM cells. Transcriptional analysis showed that BM mesenchymal stromal cells (BMSCs) were severely affected, with a reduction in cell ratio, abnormal metabolism, compromised differentiation potential, and defective hematopoiesis-supportive function, all of which were validated by functional assays. We found that ruxolitinib, a selective *JAK1/2* inhibitor, ameliorated aGVHD-related hematopoietic dysfunction through a direct effect on recipient BMSCs, resulting in improved proliferation ability, adipogenesis/osteogenesis potential, mitochondria metabolism capacity, and crosstalk with donor-derived hematopoietic stem/progenitor cells. By inhibiting the JAK2/STAT1 pathway, ruxolitinib maintained long-term improvement of aGVHD BMSC function. Additionally, ruxolitinib pretreatment in vitro primed BMSCs to better support donor-derived hematopoiesis in vivo. These observations in the murine model were validated in patient samples. Overall, our findings suggest that ruxolitinib can directly restore BMSC function via the JAK2/STAT1 pathway and, in turn, improve the hematopoietic dysfunction caused by aGVHD.

## Graphical abstract

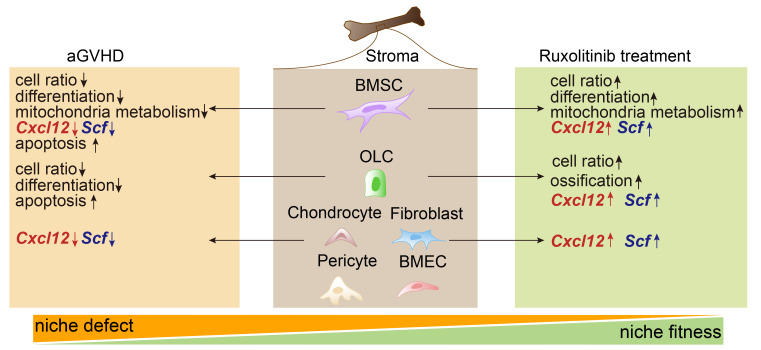

## Introduction

Allogeneic hematopoietic stem cell transplantation (allo-HSCT) is a unique therapy with curative potential for hematologic malignancies. However, acute graft-versus-host disease (aGVHD) remains one of the leading causes of morbidity and nonrelapse mortality (NRM) in the early stage after allo-HSCT (1, 2). One of the “secondary disasters” of aGVHD is the failure of donor-derived hematopoiesis, which is typically characterized by myelosuppression with an incidence of 10%–20% (3, 4). Although several risk factors have been identified, the pathophysiology of myelosuppression is still largely unclear (5). Our previous research, as well as studies by other groups, has revealed that, in addition to the direct suppression of hematopoietic primitive cells, a distorted bone marrow (BM) niche also contributes to secondary hematopoietic dysfunction (4, 6, 7). These findings highlight the importance of deciphering the extrinsic hematopoietic regulators and the BM niche in the context of aGVHD.

The BM niche is a specialized microenvironment that maintains and sustains hematopoietic stem cell (HSC) function through cellular interactions and paracrine cytokines. The niche cellular components mainly include BM mesenchymal stromal cells (BMSCs), osteolineage cells (OLCs), BM endothelial cells (BMECs), arterioles and sinusoids, sympathetic nerves, nonmyelinating Schwann cells, and megakaryocytes (8). BMSCs, which are the major cellular participants in the niche, can differentiate into other cellular components, such as osteoblasts, adipocytes, and chondrocytes (9). Located mainly in the perivascular space, BMSCs produce key niche factors, such as C-X-C motif chemokine ligand 12 (CXCL12) and stem cell factor (SCF), which support the homing and maintenance of HSCs (10). Because these nonhematopoietic BM niche cells are of recipient origin, they can be recognized and attacked as foreign by the alloreactive donor T cells when aGVHD is established (11). However, the mechanisms underlying niche remodeling in aGVHD are largely unknown.

To comprehensively assess the alterations of cellular compartments in the aGVHD niche, we performed single-cell RNA-Seq (scRNA-Seq) and constructed an atlas of niche components. In our murine aGVHD model, we observed a remarkable reduction in the BMSC ratio and function. Notably, we observed upregulation of the JAK/STAT pathway in BMSCs isolated from aGVHD hosts, making them potential targets for ruxolitinib, a selective JAK1/2 inhibitor widely used in the prophylaxis and treatment of aGVHD (12, 13). Ruxolitinib directly rescued the BMSC dysfunction caused by aGVHD by inhibiting the JAK2/STAT1 pathway, resulting in enhanced hematopoietic support capacity, differentiation potential, and mitochondrial competence. Moreover, ruxolitinib enhanced the crosstalk between BMSCs and donor-derived hematopoietic stem/progenitor cells (HSPCs) by mitochondrial transfer, in turn forming a better microenvironment for HSC engraftment. These results were also validated with clinical samples. We found the therapeutic effect of ruxolitinib on BMSCs to be long-lasting both in our aGVHD murine model and in patients. Therefore, by combining scRNA-Seq with functional assays, we revealed a mechanism underlying the effect of ruxolitinib on aGVHD niche components and broadened the BMSC-based therapy for aGVHD.

## Results

### scRNA-Seq revealed niche disruption in a murine model of aGVHD.

**Figure 1:**
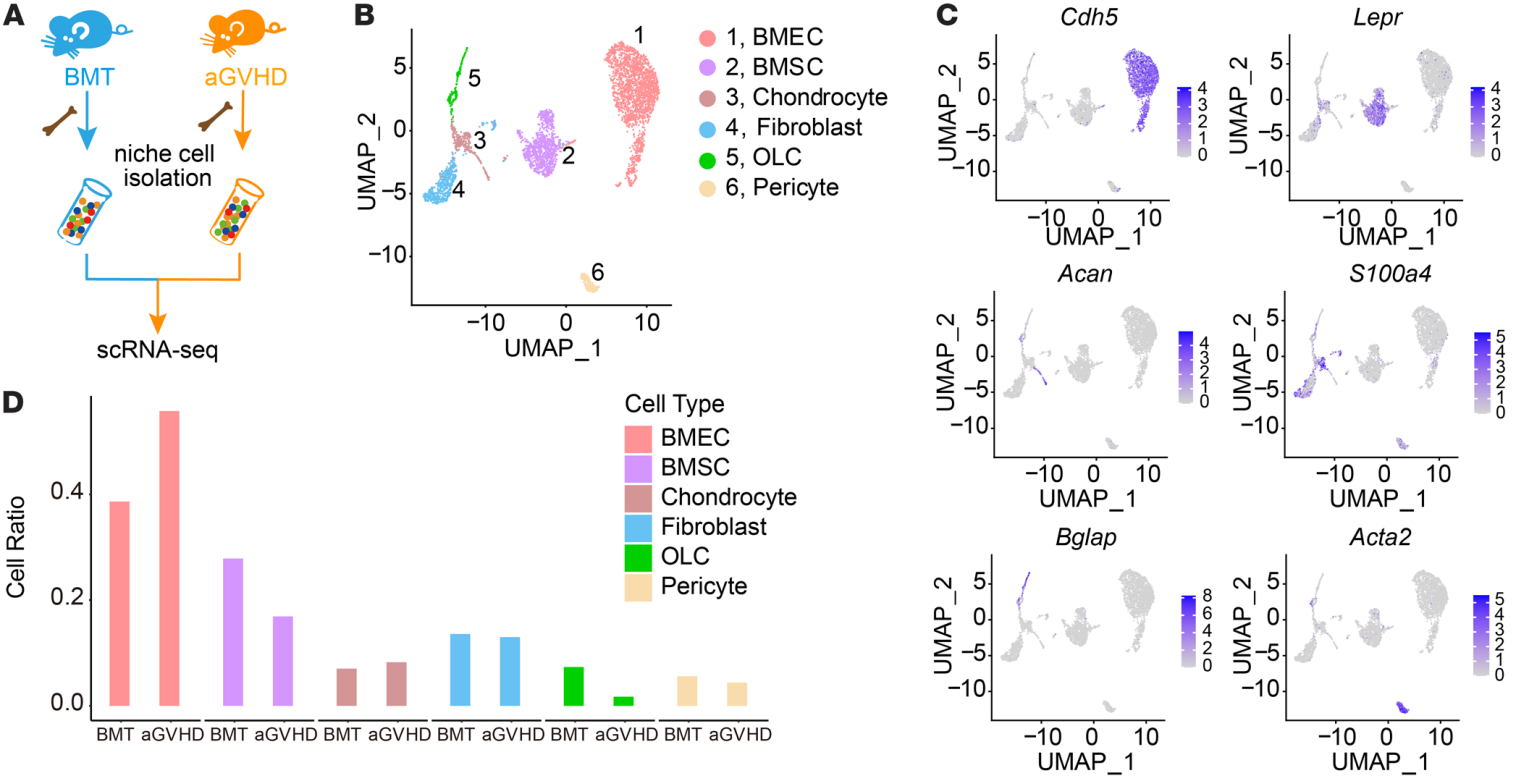
scRNA-Seq of BM niche cells. (**A**) Study procedure. BMT, *n* = 10; aGVHD, *n* = 16. (**B**) *t*-SNE map of nonhematopoietic cells from BMT and aGVHD mice. The different clusters are colored coded. (**C**) Expression of key marker genes in niche cells. (**D**) Ratio of cellular components within the BM niche of BMT and aGVHD mice.

**Figure 2:**
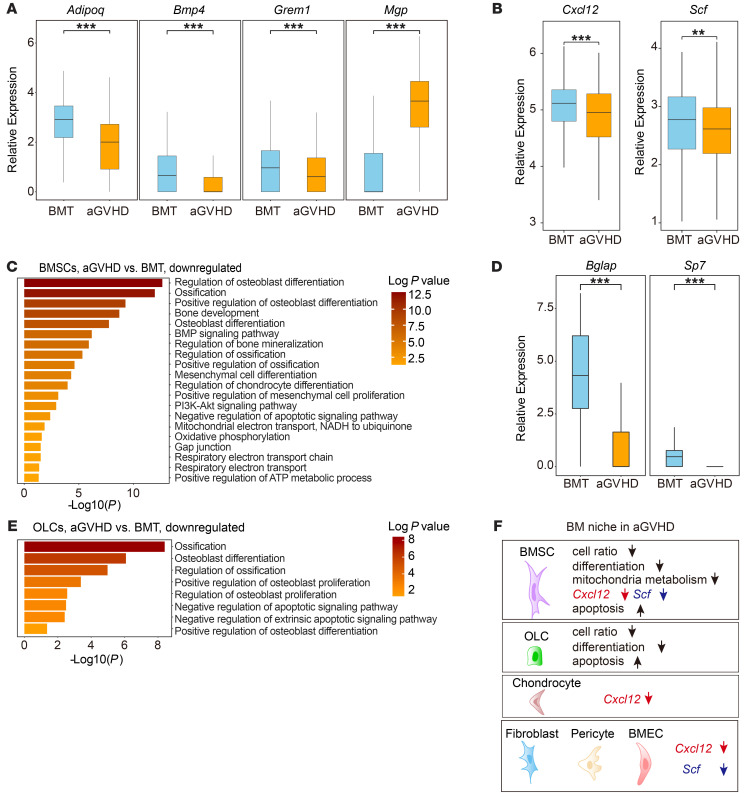
scRNA-Seq reveals BMSC disruption in a murine model of aGVHD. (**A**) Expression of differentiation-related genes in BMSCs. (**B**) Expression levels of niche factor genes in BMSCs. (**C**) Enrichment of GO terms in BMSCs. (**D**) Expression of osteoblast differentiation (*Bglap*) and maturation-related (*Sp7*) genes in OLCs. (**E**) GO term analysis of significantly downregulated genes in OLCs. (**F**) Graphical summary of niche remodeling under aGVHD conditions. **P < 0.01 and ***P < 0.001, by Wilcoxon rank-sum test to determine the significance of gene expression between the aGVHD and BMT groups (**A**, **B**, and **D**).

To dissect the alterations in the cellular composition of the aGVHD BM niche, we used a haplo-matched allo-HSCT murine model that recapitulated clinical aGVHD (7). We isolated niche cells from control (BM transplantation [BMT]) and aGVHD mice by FACS by labeling for CD45, lineage mix, and CD71 to deplete hematopoietic cells and erythrocytes, as well as for calcein acetoxymethyl ester (calcein AM) and DAPI to eliminate dead cells (Supplemental Figure 1A; supplemental material available online with this article; https://doi.org/10.1172/JCI162201DS1). These cells were collected on day 21 after transplantation and analyzed by droplet-based scRNA-Seq (10X Genomics) (Figure 1A). We partitioned all enriched cells into 17 clusters (Supplemental Figure 1B). Correlation analysis based on average gene expression and marker gene profiles distinguished nonhematopoietic cells (niche cells) from hematopoietic cells (Supplemental Figure 1, C–H) (14). Six clusters of nonhematopoietic cells were identified as follows: cluster 1, BMECs (expressing *Cdh5*); cluster 2, BMSCs (expressing *Lepr*); cluster #3, chondrocytes (expressing *Acan*); cluster 4, fibroblasts (expressing *S100a4*); cluster 5, OLCs (expressing *Bglap*); and cluster 6, pericytes (expressing *Acta2*) (Figure 1, B and C). The cell ratio of BMSCs and OLCs decreased markedly under aGVHD conditions (Figure 1D). aGVHD BMSCs presented an aberrant gene profile, including the downregulation of the differentiation-related genes *Adipoq* and *Bmp4* (15, 16) and the stemness-related gene *Grem1* (17, 18) and the upregulation of the calcification-inhibiting gene *Mgp* (19) (Figure 2A), suggesting that aGVHD impaired the stemness and differentiation potential of BMSCs. Furthermore, aGVHD disrupted niche factor (*Cxcl12* and *Scf*) expression in BMSCs (Figure 2B). Gene ontology (GO) term analysis revealed downregulation of differentiation-related pathway genes (Figure 2C) and upregulation of apoptosis-related pathway genes in aGVHD BMSCs (Supplemental Figure 1I). We noticed that aGVHD disrupted mitochondria metabolism in BMSCs, including oxidative phosphorylation (OXPHOS), respiratory electron transport chain processes, and mitochondria transfer–related pathways (phosphoinositide 3-kinase [PI3k/Akt] signaling and gap junction) (Figure 2C), suggesting that mitochondria play an important role during niche remodeling under aGVHD conditions.

We observed that other niche cellular components were also disrupted by aGVHD. Osteoblast differentiation- and maturation-related genes, critical OLC-specification genes (such as *Bglap* and *Sp7*), and niche factors were suppressed in aGVHD OLCs (Figure 2D and Supplemental Figure 1J), indicating an impairment of osteolineage cells in the aGVHD milieu. These results were consistent with the GO term analysis (Figure 2E). Although the frequency of BMECs increased in aGVHD (Figure 1D), their hematopoiesis-supportive function decreased significantly (Supplemental Figure 1J). Further subclustering revealed that aGVHD preferentially disrupted sinusoidal BMECs (Supplemental Figure 1, K–M). The expression of *Cxcl12* and *Scf* was more abundant in arteriolar BMECs and substantially inhibited by aGVHD (Supplemental Figure 1N). Additionally, aGVHD caused broad suppression of niche factor expression among fibroblasts, pericytes, and chondrocytes (Supplemental Figure 1J). These data allowed us to draft an atlas of the cellular components in the aGVHD BM niche, in which BMSCs exhibited impaired differentiation potential, defective metabolism capacity, and reduced hematopoiesis-supportive function (Figure 2F).

### BMSCs in aGVHD mice and patients with aGVHD shared similar features under aGVHD conditions.

**Figure 3:**
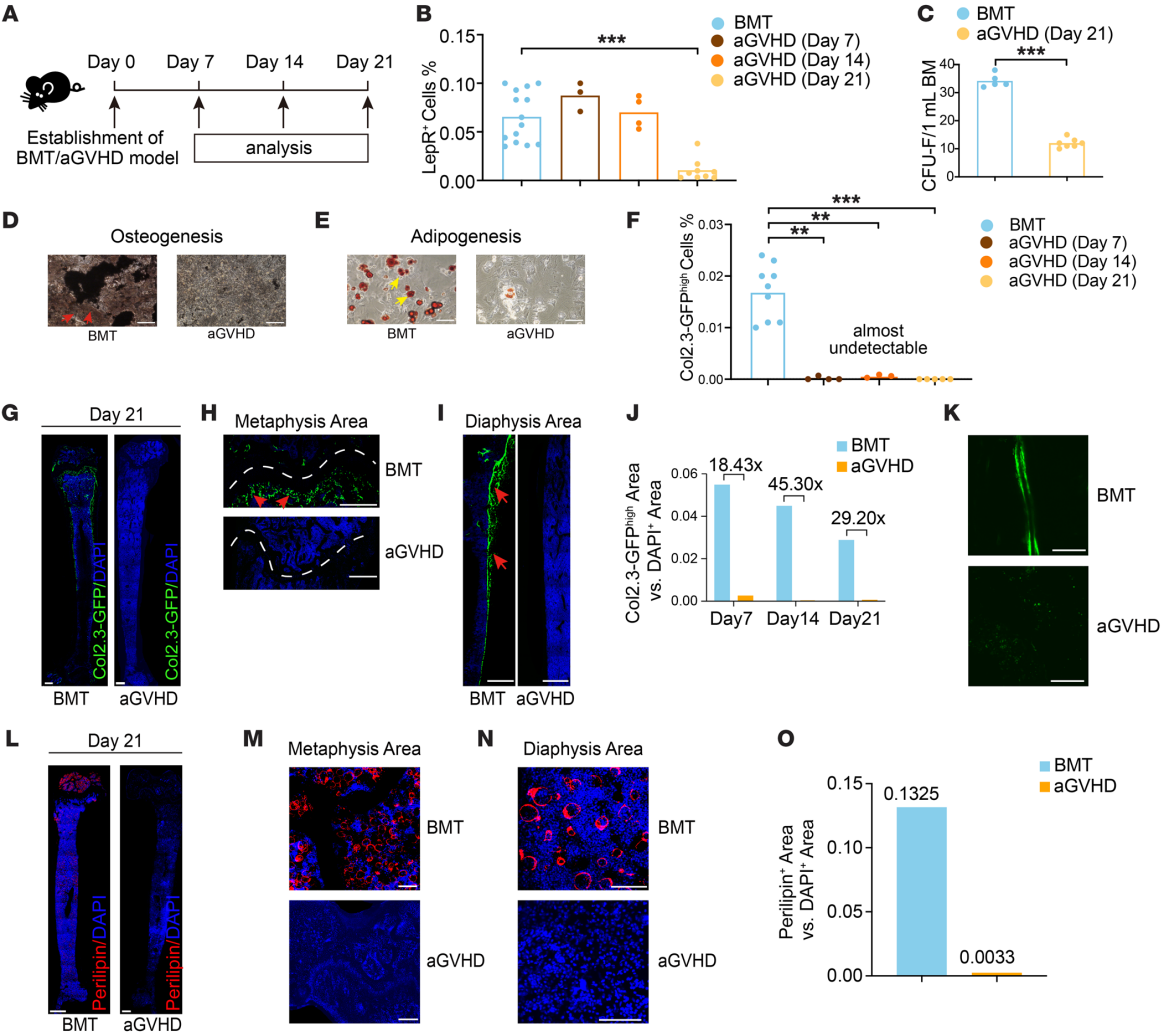
Impaired BM niche in a murine model of aGVHD. (**A**) Overview of murine aGVHD model study. (**B**) Frequency of LepR^+^ cells; *n* = 10–15 per group. (**C**) Quantitation of BMSCs by CFU-F assay; *n* = independent replicates. (**D** and **E**) Osteogenesis (**D**) and adipogenesis (**E**) of BMSCs from BMT and aGVHD mice after in vitro induction. Red arrows indicate calcium nodules (**D**), and yellow arrows indicate fatty droplets (**E**). Scale bars: 200 μm. (**F**) Quantitation of Col2.3-GFP^hi^ osteoblasts; *n* = 10–15 per group. (**G**) Immunofluorescence images of femurs at day 21; three independent replicates. Scale bars: 500 μm. (**H** and **I**) High-magnification views of metaphysis areas (**H**) and diaphysis area (**I**). White dotted lines represent the growth plate, and red arrows indicate Col2.3-GFP^hi^ osteoblasts. Scale bars: 200 μm. (**J**) Quantitative analysis of Col2.3-GFP^hi^ area versus DAPI^+^ area in BMT and aGVHD mice. Numbers above columns represent the fold change compared with BMT mice; *n* = 3 independent replicates. (**K**) Images of calcein-stained femurs; 3 independent replicates. Scale bars: 50 μm. (**L**) Immunofluorescence images of adipocytes; *n* = independent replicates. Scale bars: 500 μm. (**M** and **N**) High-magnification views of metaphysis (**M**) and diaphysis (**N**) areas. Scale bars: 200 μm. (**O**) Quantitative analysis of perilipin^+^ area versus DAPI^+^ area; *n* = independent replicates. **P < 0.01 and ***P < 0.001, by 1-way ANOVA followed by an unpaired, 2-tailed *t* test (**B** and **F**), and unpaired, 2-tailed *t* test (**C**).

To validate our transcriptomic analysis, we next conducted functional assays in a haplo-matched aGVHD murine model (Figure 3A). The absolute number of niche cells decreased longitudinally from day 7 to day 21 in aGVHD mice (Supplemental Figure 2A). Leptin receptor^+^ (LepR^+^) BMSCs in aGVHD mice exhibited a 36-fold reduction compared with their counterparts in the BMT group by day 21 (Figure 3B and Supplemental Figure 2B). aGVHD BMSCs also showed impaired colony-forming ability (Figure 3C and Supplemental Figure 2, C and D), reduced osteogenic (Figure 3D, red arrows) and adipogenic differentiation potential (Figure 3E, yellow arrows). Bulk RNA-Seq and quantitative real-time PCR (qPCR) showed downregulation of genes related to stemness (*Lepr*, *Grem1*) and differentiation (*Adipoq*, *PPARG*, *Runx2*, *Alpl*, *Col1a1*, and *Col2a1*) in aGVHD BMSCs (Supplemental Figure 2, E and F).

To investigate differences in osteolineage dynamics over different stages of aGVHD, we generated Col2.3-GFP reporter–transgenic mice, which specifically expressed GFP in osteoblasts and osteocytes (20). We observed a striking decrease in osteoblasts (Col2.3-GFP^hi^) from the early (day 7) to late (day 21) stages of aGVHD, with a 28-fold reduction compared with the BMT group (Figure 3F and Supplemental Figure 2G). Femurs from aGVHD mice also showed much weaker Col2.3-GFP signals in regions of metaphysis and diaphysis (Figure 3, G–I, and Supplemental Figure 2, H–J), which was confirmed by the reduction in the proportion of the Col2.3-GFP^hi^ area (Figure 3J). Calcein labeling showed that bone formation was also severely restricted in aGVHD mice (Figure 3K). These findings suggested that aGVHD severely impaired osteolineage dynamics and bone formation.

In contrast to the diffuse distribution of adipocytes in the femurs of BMT mice, adipocytes in aGVHD mice were almost undetectable (Figure 3, L–N). We observed a 40-fold decrease in adipocytes in aGVHD mice compared with BMT mice (Figure 3O). However, oral administration of 2 major isoforms of the PPARγ agonists rosiglitazone (thiazolidinedione) and GW1929 (non-thiazolidinedione) did not significantly improve the survival of aGVHD mice (Supplemental Figure 3, A and B), although GW1929 administration increased adipocyte frequency in aGVHD mice (Supplemental Figure 3C). These results indicated that rescuing adipocytes alone was not sufficient to improve the survival of aGVHD.

**Figure 4:**
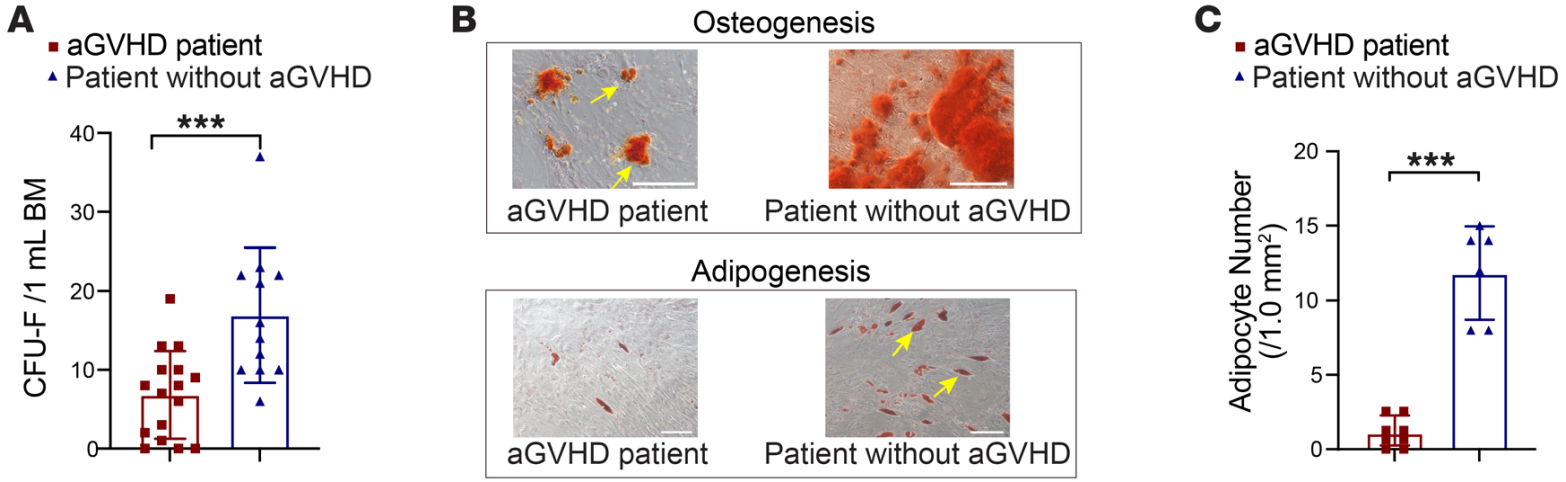
Impaired BM niche in patients with aGVHD. (**A**) Colony-forming assay of BMSCs from patients with aGVHD and allo-HSCT patients without aGVHD; *n* = 11–18 per group. (**B**) Representative osteogenesis (upper) and adipogenesis (lower) results; *n* = independent replicates. Arrows indicate calcium nodules (upper) and fatty droplets (lower). Scale bars: 200 μm. (**C**) Quantification of adipocyte numbers after in vitro induction; *n* = 6–8 per group. ***P < 0.001, by unpaired, 2-tailed *t* test (**A** and **C**).

To validate our observations in human samples, we collected the BM aspirates from patients with or without aGVHD (Table 1). The median platelet count (*P* = 0.0062) and hemoglobin level (*P* *<* 0.0001) in the aGVHD group were significantly lower compared with those of their counterparts in the non-aGVHD group. BMSCs from patients with aGVHD were defective in colony-forming ability (Figure 4A) and adipogenic and osteogenic differentiation potential (Figure 4, B and C). These data indicated that BMSCs from aGVHD mice and from patients with aGVHD exhibited similar impairment under aGVHD conditions.

### Ruxolitinib directly rescued BMSC function in aGVHD.

**Figure 5:**
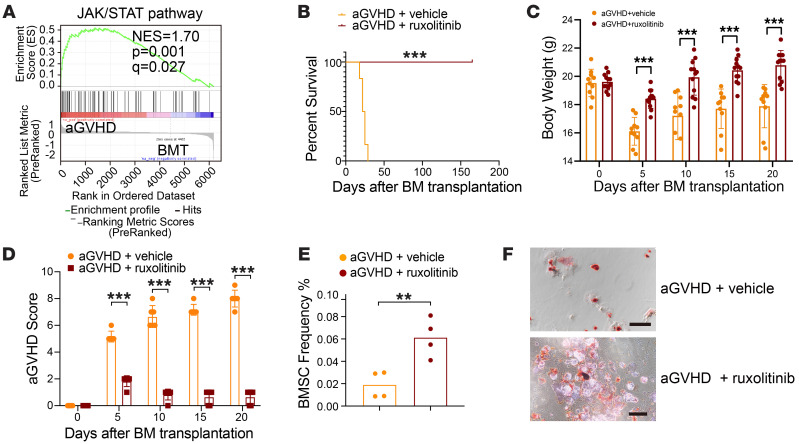
Ruxolitinib enhances BMSC function in aGVHD mice. (**A**) Kyoto Encyclopedia of Genes and Genomes (KEGG) pathway enrichment of JAK/STAT signaling in BMSCs isolated from BMT and aGVHD mice; *n* = 30 per group. NES, normalized enrichment score. (**B**–**D**) Survival curve (**B**), body weight (**C**), and aGVHD score (**D**) for aGVHD mice after vehicle or ruxolitinib treatment (30 mg/kg in solvent, oral administration, twice a day); *n* = 6 per group. (**E**) Alterations of LepR^+^ BMSC frequency after ruxolitinib treatment; *n* = 4 per group. (**F**) Osteogenic differentiation assay of BMSCs from ruxolitinib- or vehicle-treated aGVHD mice; *n* = 3 independent replicates. Scale bars: 200 μm. **P < 0.01 and ***P < 0.001, by log-rank test (**B**), 1-way ANOVA followed by unpaired, 2-tailed *t* test (**C** and **D**), and unpaired, 2-tailed *t* test (**E**).

In our transcriptomic analysis, we noticed a significant upregulation of the JAK/STAT pathway in aGVHD BMSCs (Figure 5A), which led us to hypothesize that BMSCs could be a potential target of ruxolitinib, a selective *JAK1/2* inhibitor approved by the FDA for the treatment of steroid-refractory aGVHD (21). In aGVHD mice, oral administration of ruxolitinib (10 mg/kg or 30 mg/kg, twice a day for 20 days) resulted in a lifespan extension of 50 days and 150 days, respectively (Figure 5B and Supplemental Figure 4A). Notably, treatment with 30 mg/kg ruxolitinib markedly ameliorated body weight loss and reduced the disease score in aGVHD mice (Figure 5, C and D). We also observed the recovery of hematopoietic health based on increased BM cellularity, HSC reconstitution, and B cell frequency (Supplemental Figure 4B). Furthermore, the effect of ruxolitinib persisted out to 150 days after transplantation (130 days after ruxolitinib treatment), suggesting that this effect was not transient (Supplemental Figure 4, C and D). LepR^+^ BMSCs from aGVHD mice exhibited a greater than 3-fold increase after ruxolitinib treatment compared with BMSCs from matched controls (Figure 5E). BMSCs from ruxolitinib-treated mice generated detectable calcium nodules after induction of osteogenesis, whereas those from the vehicle-treated group did not (Figure 5F), implying an enhanced osteogenic potential of aGVHD BMSCs after ruxolitinib treatment. These results suggested that ruxolitinib directly rescued BMSC function in aGVHD and may also be a potential therapeutic target for the aGVHD niche.

**Figure 6:**
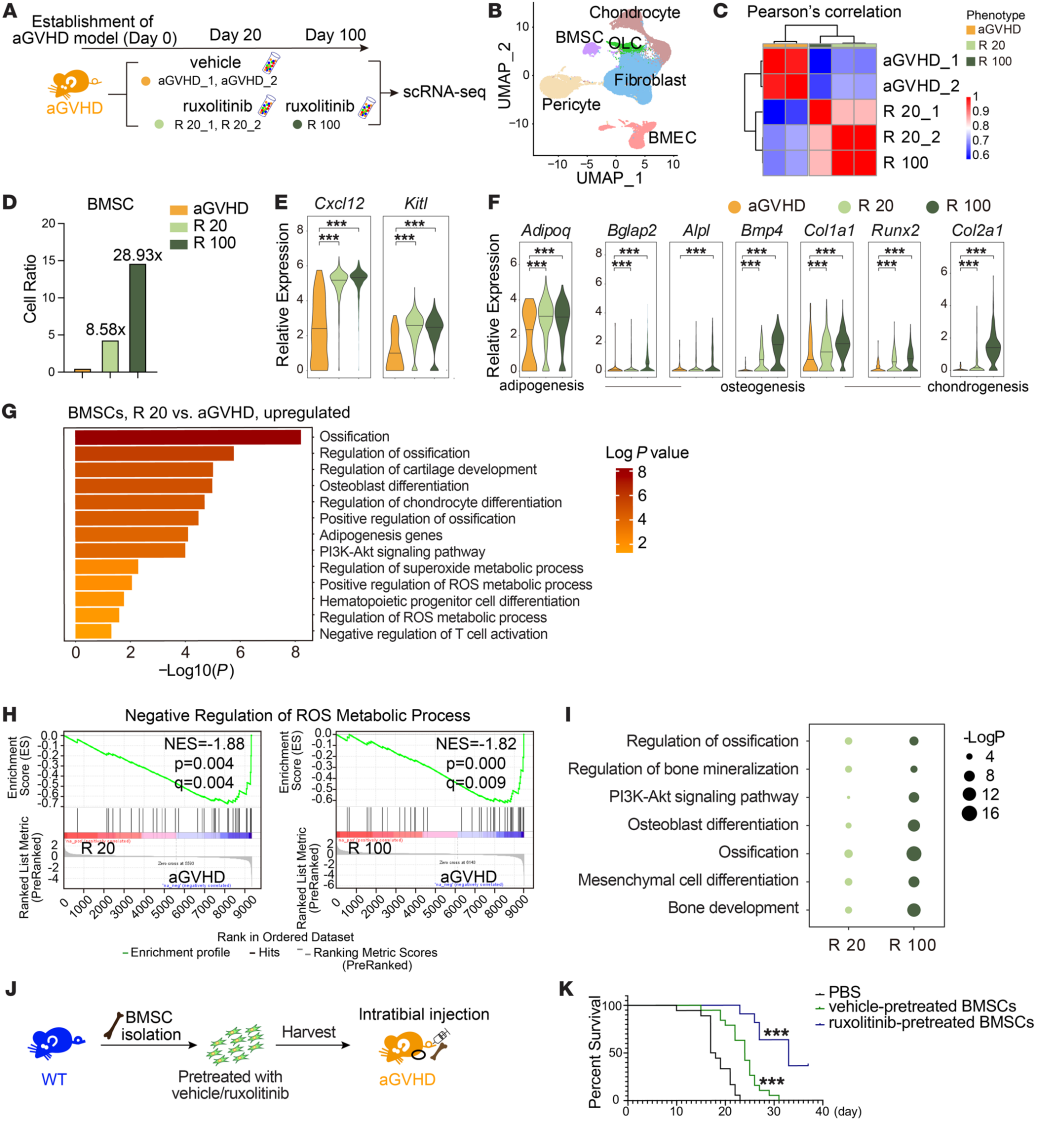
scRNA-Seq shows long-term protective effects of ruxolitinib on aGVHD BMSCs. (**A**) Procedure for scRNA-Seq during long-term follow-up after ruxolitinb treatment. aGVHD, *n* = 16 per sample; R 20, *n* = 20 per sample; R 100, *n* = 10. (**B**) *t*-SNE map of niche cells. (**C**) Pearson’s correlation between the average gene expression profiles of samples. (**D**) BMSC ratio during long-term follow-up after ruxolitinb treatment. (**E** and **F**) Violin plots of expression levels of niche factor– (**E**) and differentiation-related (**F**) genes in BMSCs. (**G**) GO term enrichment of upregulated genes in ruxolitinib-treated aGVHD BMSCs. (**H**) GO term enrichment of ROS metabolic process–related pathway. (**I**) Common features of BMSCs among samples at different time points. (**J** and **K**) Procedure (**J**) and survival (**K**) after intratibial BMSC injection (cultured BMSCs, pretreated with vehicle or ruxolitinib); *n* = 10 per group. ***P < 0.001, by Wilcoxon rank-sum test (**E** and **F**) and log-rank test (**K**). R 20, day 20 after transplantation (12 hours after ruxolitinib treatment); R 100, day 100 after transplantation (day 80 after ruxolitinib treatment).

Because of the low frequency of BMSCs in individuals with aGVHD, we performed scRNA-Seq on niche cells (CD45^−^lineage^−^CD71^−^calcein AM^+^DAPI^−^) from vehicle- and ruxolitinib-treated aGVHD mice (day 20 after transplantation [12 hours after ruxolitinib treatment] and day 100 after transplantation [80 days after ruxolitinib treatment]) to investigate the long-term remodeling of BMSCs (Figure 6A). After removal of contaminating hematopoietic cells (Supplemental Figure 4, E and F), unsupervised clustering partitioned the stroma into 6 clusters comprising BMSCs, OLCs, BMECs, pericytes, chondrocytes, and fibroblasts (Figure 6B) according to marker gene expression patterns (Supplemental Figure 4G). Niche cells exposed to ruxolitinib exhibited a transcriptional phenotype different from those of the vehicle-treated controls (Figure 6C). Cell ratio analysis revealed that BMSCs were markedly boosted after ruxolitinib treatment, which was consistent with the results of our functional assay (Figure 6D). Enhanced hematopoiesis-supportive function and differentiation potential were achieved for BMSCs after ruxolitinib treatment (Figure 6, E and F). Interestingly, ruxolitinib treatment significantly upregulated mitochondria metabolism–related genes, such as those associated with OXPHOS, ROS, and metabolic processes in aGVHD BMSCs (Figure 6G), which was validated by elevated MitoSox levels in BMSCs derived from ruxolitinib-treated mice (Supplemental Figure 4H). In addition, upregulation of the mitochondria transfer–related pathway (PI3k/Akt signaling) (Figure 6G) and downregulation of apoptosis-related pathways were observed in ruxolitinib-treated aGVHD BMSCs (Supplemental Figure 4I). In both the short- and long-term follow-up analyses after ruxolitinib treatment, we observed enhanced ROS metabolism in BMSCs (Figure 6H), indicating that ruxolitinib maintained relatively high mitochondria metabolism for a long period. Additionally, BMSCs shared common features from day 20 to day 100 after transplantation, including upregulation of differentiation potential (especially osteogenesis) and the PI3k/Akt pathway (Figure 6I). Our transcriptomic analysis suggested that ruxolitinib restored niche fitness, which was disrupted by aGVHD and provided long-term protective effects for BMSCs in the aGVHD milieu.

To investigate the function of ruxolitinib-treated BMSCs in vivo, aGVHD mice were injected with primary or cultured BMSCs (Figure 6J and Supplemental Figure 5A). Intravenous injection of BMSCs did not prolong the survival of aGVHD mice (Supplemental Figure 5, B and C). However, intratibial injection of 1 × 10^6^ cultured BMSCs significantly prolonged the lifespan of aGVHD mice (23 days vs. 17 days; Figure 6K). Ruxolitinib enhanced the colony-forming ability of BMSCs in a dose-dependent manner in vitro (Supplemental Figure 5D); therefore, we preconditioned BMSCs with 0.5 μM ruxolitinib before the injection. Intratibial injection of ruxolitinib-pretreated BMSCs further improved the survival of aGVHD mice compared with those administered with vehicle-treated BMSCs (Figure 6K), indicating that ruxolitinib pretreatment in vitro primed BMSCs to function better in vivo. Jointly, these data demonstrated that ruxolitinib directly restored BMSC function, resulting in enhanced differentiation potential, hematopoiesis-supportive function, and metabolism capacity.

### Ruxolitinib enhanced hematopoietic regeneration by promoting mitochondria transfer from recipient BMSCs to donor-derived HSPCs.

**Figure 7:**
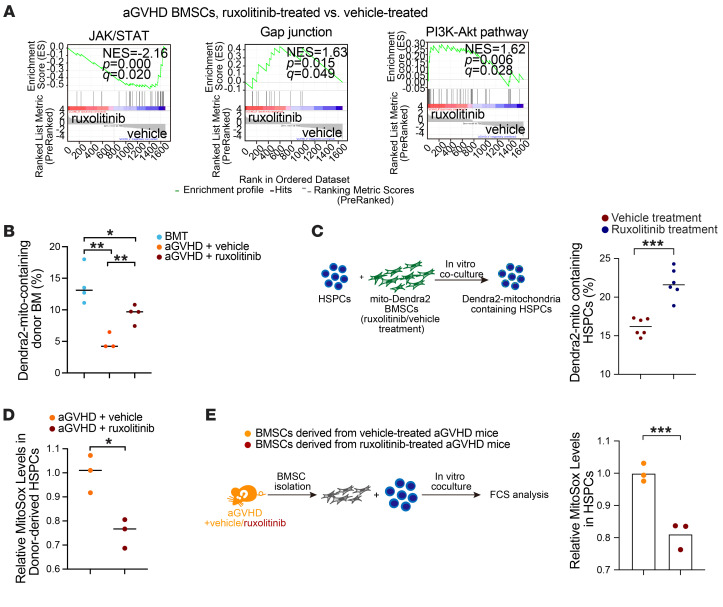
Ruxolitinib enhances hematopoietic regeneration by promoting mitochondria transfer from BMSCs to donor-derived HSPCs. (**A**) Gene set enrichment analysis (GSEA) of the JAK/STAT pathway and critical mitochondria transfer–related pathway in vehicle- or ruxolitinib-treated aGVHD BMSCs. (**B**) Mitochondria transfer from recipient BMSCs to donor BM cells (represented by the percentage of transplanted BM cells containing host BMSC–derived Dendra2^+^ mitochondria [mito-containing]); *n* = 3–4 per group. (**C**) Schematic illustration and percentage of mitochondria transfer from BMSCs to HSPCs after in vitro coculturing; *n* = 6 per group. (**D**) Relative MitoSox levels in donor-derived HSPCs after transplantation into vehicle- or ruxolitinib-treated aGVHD recipients; *n* = 3 per group. (**E**) Strategy for in vitro coculture and relative MitoSox levels in HSPC after coculture with BMSC derived from vehicle- or ruxolitinib-treated aGVHD mice; *n* = 3 per group. *P < 0.05, **P < 0.01, and ***P < 0.001, by 1-way ANOVA followed by unpaired, 2-tailed *t* test (**B**) and unpaired, 2-tailed *t* test (**C**–**E**).

We noticed that aGVHD mouse–derived BMSCs treated with ruxolitinib in vitro showed downregulation of the JAK/STAT pathway and upregulation of gap junction–related genes and the PI3k/Akt pathway (Figure 7A), implying the activation of mitochondria transfer. We hypothesized that ruxolitinib enhanced the ability of recipient BMSCs to create a favorable microenvironment for donor cells by modulating mitochondria transfer between these 2 cell populations. Therefore, we used mito-Dendra2 reporter mice to track the mitochondrial dynamics. Oral administration of ruxolitinib increased mitochondria transfer from recipient BMSCs to donor-derived BM cells in aGVHD mice (Figure 7B). When cocultured with ruxolitinib-pretreated BMSCs, the proportion of Dendra2-mitochondria containing HSPCs was higher than that in HSPCs cocultured with vehicle-pretreated BMSCs (Figure 7C), suggesting that ruxolitinib promoted mitochondria transfer from BMSCs to HSPCs. When exposed to a ruxolitinib-treated niche, donor-derived HSPCs exhibited lower ROS levels than did those exposed to an untreated niche (Figure 7D). When isolated and cocultured with HSPCs in vitro, ruxolitinib-pretreated aGVHD BMSCs also decreased the ROS levels in HSPCs (Figure 7E), which may have contributed to hematopoietic engraftment. These data indicated that ruxolitinib enhanced the ability of BMSCs to create a favorable microenvironment for HSC maintenance and engraftment.

### Ruxolitinib directly modulated aGVHD BMSC function by inhibiting the JAK2/STAT1 pathway.

**Figure 8:**
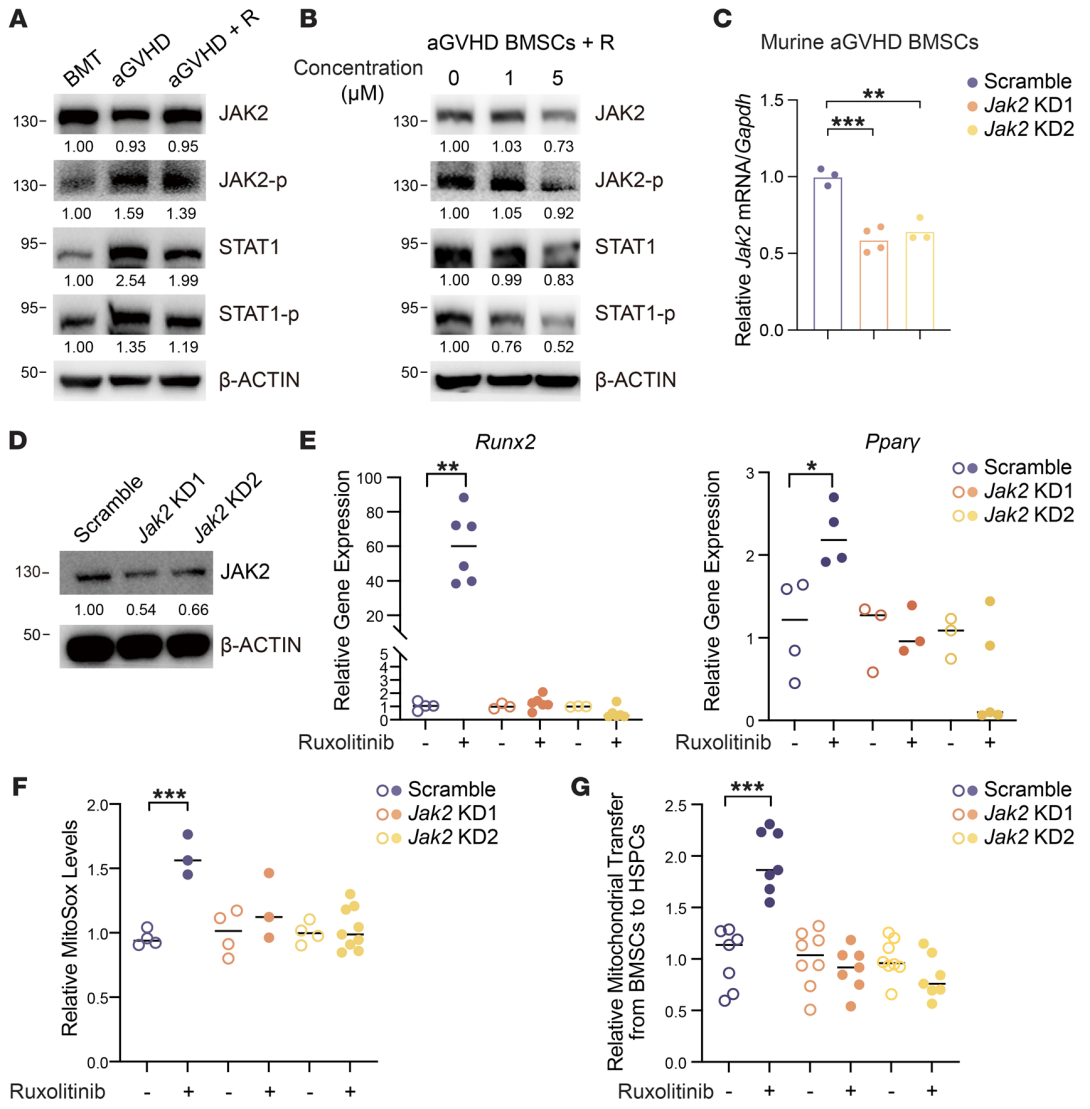
Ruxolitinib directly modulates aGVHD BMSC function by inhibiting the JAK2/STAT1 pathway. (**A**) JAK2 and STAT1 and their phosphorylation (p) levels in BMSCs derived from BMT, aGVHD, and ruxolitinib-treated (R) aGVHD mice. BMT, *n* = 10; aGVHD, *n* = 15; aGVHD+R, ruxolitinib-treated aGVHD mice, *n* = 10. Numbers indicate the fold change of protein levels normalized to BMT. (**B**) Inhibition of JAK2/STAT1 pathway in aGVHD BMSCs after incubation with different concentrations of ruxolitinib for 24 hours in vitro; *n* = 5. Numbers indicate the fold change of protein levels normalized to control. (**C** and **D**) *Jak2* mRNA (**C**) and protein (**D**) levels in aGVHD BMSCs after transfection with scrambled or *Jak2* shRNA. Numbers indicated the fold change of protein levels normalized to scrambled shRNA (**C**). (**E**) Expression of osteogenesis- (*Runx2*) and adipogenesis-related (*Pparg*) genes in scrambled or *Jak2*-deficient BMSCs after ruxolitinib treatment; *n* = 3–6 per group. (**F**) Response of BMSC mitochondria metabolism to ruxolitinib after *Jak2* knockdown; *n* = 3–9 per group. (**G**) Relative mitochondria transfer from *Jak2*-deficient BMSCs to HSPCs after vehicle or ruxolitinib treatment; *n* = 7–8 per group. *P < 0.05, **P < 0.01, and ***P < 0.001, by 1-way ANOVA followed by unpaired, 2-tailed *t* test (**E**–**G**). KD1, knockdown 1 (shRNA 1); KD2, knockdown 2 (shRNA 2).

To clarify the molecular mechanism underlying the pharmacological effects of ruxolitinib, we evaluated the levels of critical proteins in the JAK/STAT pathway. We found that aGVHD induced phosphorylation of JAK2 and STAT1 in BMSCs, which was inhibited by ruxolitinib (Figure 8A), while only mild alterations were observed in JAK1 and other members of the STAT family (Supplemental Figure 6, A and B). The selective JAK2 inhibitor fedratinib elevated expression of differentiation-related genes (*Ppar*γ and *Col1a1*) and the niche factor *Cxcl12*, whereas the selective JAK1 inhibitor itacitinib did not (Supplemental Figure 6C), indicating that ruxolitinib mediated its pharmacological effects via the JAK2/STAT1 pathway. We also confirmed the suppression of JAK2/STAT1 by in vitro treatment of aGVHD BMSCs with different concentrations of ruxolitinib (Figure 8B). We then used lentiviral shRNA to knock down *Jak2* in aGVHD BMSCs. The knockdown efficiency was validated by qPCR (Figure 8C) and Western blotting (Figure 8D). Ruxolitinib did not enhance osteogenesis or adipogenesis (Figure 8E) or mitochondria metabolism (Figure 8F) or mitochondrial transfer to cocultured HSPCs (Figure 8G) in *Jak2-*deficient aGVHD BMSCs. These data demonstrated that ruxolitinib directly primed aGVHD BMSC function by inhibiting the JAK2/STAT1 pathway.

### Ruxolitinib improved BMSC function in patients with aGVHD.

**Figure 9:**
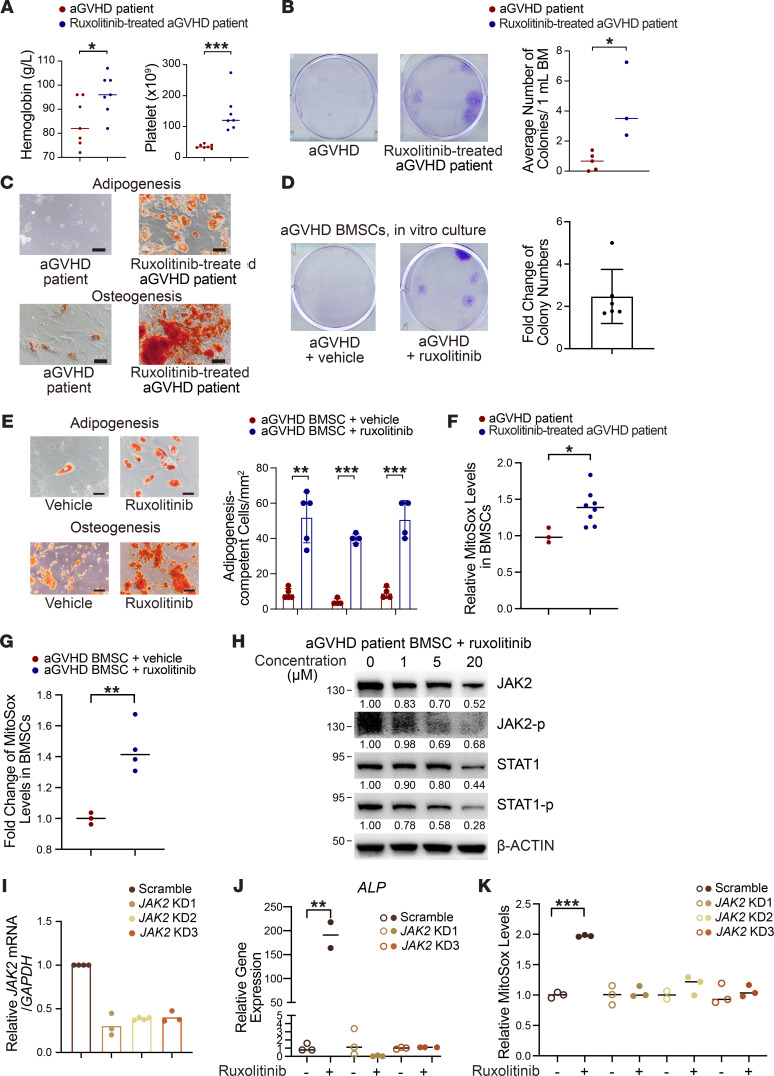
Ruxolitinib enhances BMSC function in patients with aGVHD via the JAK2/STAT1 pathway. (**A**) Hemoglobin levels and platelet counts in aGVHD patients with or without ruxolitinib treatment; *n* = 7 per group. (**B** and **C**) CFU-F assay (**B**) and adipogenesis and osteogenesis (**C**) of BMSCs isolated from patients with aGVHD with or without ruxolitinib treatment; *n* = 3–5 per group. Scale bars: 100 μm. (**D** and **E**) In vitro CFU-F ability (**D**) and differentiation potential (**E**) of aGVHD BMSCs treated with vehicle or ruxolitinib; *n* = 4–6 per group. Scale bars: 100 μm. (**F**) Relative cellular ROS levels in BMSCs isolated from aGVHD patients with or without ruxolitinib treatment; *n* = 3–8 per group. (**G**) Relative cellular ROS levels in aGVHD BMSCs after vehicle or ruxolitinib treatment in vitro; *n* = 3–4 per group. (**H**) Inhibition of the JAK2/STAT1 pathway in aGVHD BMSCs after incubation with ruxolitinib (1, 5, and 20 μM) for 24 hours in vitro. Numbers indicate the fold change of protein levels normalized to control. (**I**) *JAK2* mRNA levels in aGVHD BMSCs after transfection with scrambled or *JAK2* shRNAs. (**J** and **K**) Response of BMSC osteogenesis (**J**) and mitochondria metabolism (**K**) to ruxolitinib treatment after *JAK2* knockdown in BMSCs derived from patients with aGVHD; *n* = 3 per group. *P < 0.05, **P < 0.01, and ***P < 0.001, by 1-way ANOVA followed by unpaired, 2-tailed *t* test (**J** and **K**) and by unpaired, 2-tailed *t* test (**A**, **B**, **E**, **F**, and **G**).

To investigate whether ruxolitinib treatment directly improves BMSC function in patients with aGVHD, we collected BM aspiration samples from patients with aGVHD before and after ruxolitinib treatment. The clinical information on the patients is summarized in Supplemental Table 1. Ruxolitinib promoted hematopoietic recovery in patients with aGVHD (Figure 9A). In accordance with our findings in the aGVHD murine model, ruxolitinib increased the number of proliferation-competent BMSCs (Figure 9B) and enhanced the adipogenesis and osteogenesis potential of BMSCs in aGVHD patients (Figure 9C). Incubation with ruxolitinib in vitro resulted in a greater than 2.0-fold increase in aGVHD BMSC colonies compared with BMSCs incubated with the vehicle (Figure 9D). This increase was accompanied by improvement in adipogenic and osteogenic differentiation potentials (Figure 9E). Notably, ruxolitinib treatment elevated the mitochondria metabolism of BMSCs in patients with aGVHD (Figure 9F). Compared with aGVHD BMSCs treated with vehicle in vitro, those treated with ruxolitinib also showed elevated ROS levels (Figure 9G). In accordance with our findings in the aGVHD murine model, ruxolitinib inhibited activation of the JAK2/STAT1 pathway in aGVHD patient–derived BMSCs (Figure 9H). Following shRNA-mediated knockdown of *JAK2* (Figure 9I), ruxolitinib no longer enhanced osteogenesis (Figure 9J) or mitochondria metabolism of aGVHD BMSCs (Figure 9K). In our follow-up of patients up to 6 months after ruxolitinib administration (Supplemental Table 2), we were surprised to observe continuously enhanced colony-forming ability (Supplemental Figure 7A), osteogenesis (Supplemental Figure 7B), adipogenesis (Supplemental Figure 7, C and D), and mitochondria metabolism (Supplemental Figure 7E) of BMSCs. These data indicated that ruxolitinib acted directly to salvage BMSC function via the JAK2/STAT1 pathway and maintained long-term therapeutic effects.

## Discussion

Accumulating evidence has suggested that the BM niche serves as a crucial therapeutic target in aGVHD. However, the biological abnormalities underlying the BM niche in aGVHD are unclear. For the first time to our knowledge, we have constructed an atlas to illustrate the cellular components of the BM niche in aGVHD models, providing a more comprehensive and precise understanding of BM niche remodeling under aGVHD conditions. Through the integration of transcriptional analysis and functional assays, we systematically identified several remodeling events in the aGVHD BM niche. Specifically, we found that (a) the ratio of cellular components, particularly BMSCs and OLCs, was significantly reduced; (b) BMSCs exhibited impaired differentiation potential, mitochondria metabolism, and crosstalk with donor-derived HSPCs; (c) OLCs displayed compromised differentiation and maturation ability; and (d) the hematopoietic support capacity of most niche cells was markedly decreased.

Ruxolitinib has been successfully applied in the treatment of acute and chronic GVHD in recent years (22, 23). The potent therapeutic effect of ruxolitinib was also demonstrated by our clinical and aGVHD model observations. For the first time to our knowledge, we have revealed the direct impact of ruxolitinib on BMSCs via the JAK2/STAT1 pathway in addition to its well-known systemic immunomodulatory function (13). Our findings were substantiated by several lines of evidence: (a) RNA-Seq analysis demonstrated that both in vivo and in vitro exposure to ruxolitinib downregulated the JAK/STAT pathway in aGVHD BMSCs (Figure 5A and Figure 7A); (b) Western blotting experiments revealed that aGVHD-induced activation of the JAK2/STAT1 pathway was effectively attenuated by ruxolitinib treatment (Figure 8, A and B, and Figure 9H); and (c) *Jak2* knockdown abrogated the therapeutic effect of ruxolitinib on aGVHD BMSCs (Figure 8, E–G, and Figure 9, J and K). The revelation of the direct protective effects of ruxolitinib on BMSCs has pivotal clinical significance for several reasons. First, JAKs function as crucial regulators of immune cells and play pivotal roles in all phases of aGVHD pathogenesis (24). The restoration of BMSC functions by ruxolitinib indicates that BMSCs could be a direct sensor of pathological signals. Second, we discovered that ruxolitinib restored BMSC metabolism, particularly ROS production. Previous studies have shown that JAK/STAT blockade by inhibitors such as tofacitinib significantly enhanced oxidative phosphorylation, ATP production, maximal respiratory capacity, and respiratory reserve in rheumatoid arthritis synovial fibroblasts (25). However, a functional link between JAK/STAT signaling and BMSC metabolism in aGVHD has yet to be established. We believe our findings have opened a new avenue of research, suggesting that metabolic agents could potentially serve as a viable option for microenvironment-based therapy.

The crosstalk between stromal cells and HSCs has been recognized as an important extrinsic mechanism of HSC regulation. Activated BMSCs were found to abrogate the ROS levels of target cells via mitochondria transfer, with reduced apoptosis and cell death (26), conferring survival benefits for recipient cells (27). One potential mechanism is that ROS-induced oxidative stress regulates the opening of connexin channels mediated by PI3K activation and the transfer of mitochondria from BMSCs to HSCs (28). To our knowledge, the phenomenon of mitochondria transfer in the context of aGVHD has not been investigated. In our study, we demonstrated that ruxolitinib treatment upregulated the PI3k/Akt pathway in BMSCs and promoted the transfer of functional mitochondria from BMSCs to donor-derived HSCs, thereby conferring a protective effect in aGVHD. When cocultured with BMSCs that were preexposed to ruxolitinib, c-Kit^+^ HSPCs exhibited lower cellular ROS levels. Previous studies have suggested that high levels of cellular ROS in HSPCs can lead to defects in cell-cycle quiescence, disrupted HSPC-osteoblastic niche interactions, and, ultimately, HSC exhaustion (29–31). Conversely, HSCs with low ROS levels are thought to have better long-term repopulating capacity (32). Hence, ruxolitinib treatment helps to maintain BMSC function and fosters a favorable microenvironment for donor-derived hematopoiesis, which in turn provides a supportive niche for HSCs and contributes to their mitochondrial fitness. These data indicate that ruxolitinib acts directly on BMSCs and rescues their function, representing a noncanonical pathway that partially contributes to the mechanisms underlying the therapeutic effects of ruxolitinib in aGVHD.

BMSCs possess trophic, homing/migration and immunomodulatory functions that have been demonstrated both in vitro and in vivo (33). However, the key issue of BMSCs as second-line aGVHD therapy is that the clinical outcome can be unpredictable. Therefore, there remains a pressing need to optimize BMSC efficiency in aGVHD therapy. In light of the excellent tolerability, efficacy, and safety profile of ruxolitinib observed in clinical trials (22, 34), as well as its direct effects on BMSCs, we consider ruxolitinib to be an ideal candidate for in vitro pretreatment of BMSCs to prime their function in vivo. The findings of our study have important clinical implications, as they shed light on the mechanisms by which aberrant signals from factors such as cytokines and cell-cell interactions within the aGVHD microenvironment can compromise the immunomodulatory and multilineage differentiation potential of adoptively transferred mesenchymal stem/stromal cells (MSCs). By pretreating BMSCs with ruxolitinib, we were able to tune down these aberrant signals, thereby preserving their function in the aGVHD milieu. Our data suggest that administration of ruxolitinib-pretreated MSCs holds great promise for improving hematopoietic dysfunction in aGVHD. Notably, when ruxolitinib-pretreated BMSCs were injected topically, we observed that the megakaryocyte-erythroid progenitor (MEP) frequency in BM was also boosted, implying that functionally rescued aGVHD BMSCs primed preliminary hematopoietic cells directly. Since MEPs give rise to platelets (35), we believe that BMSC injection together with ruxolitinib may overcome the potential risk of thrombocytopenia caused by ruxolitinib (22).

In conclusion, our data provide a comprehensive cellular and molecular landscape of the aGVHD BM niche. We demonstrate that ruxolitinib directly rescued aGVHD BMSC function by inhibiting the JAK2/STAT1 pathway, thereby ameliorating hematopoietic dysfunction secondary to aGVHD. These findings provide insights into the mechanisms underlying the therapeutic effects of ruxolitinib in aGVHD.

## Methods

### Patients.

To investigate BM niche alterations in aGVHD, 44 patients (34 patients with aGVHD and 10 without aGVHD) who received allo-HSCT at the Institute of Hematology and Blood Diseases Hospital (Tianjin, China) and the Shanghai Institute of Hematology, National Research Center for Translational Medicine (Shanghai, China) were enrolled between September 2018 and September 2021. The patient transplantation procedure used has been described previously (36, 37). Four patients (9.1%) received HLA-matched and -related grafts, 38 patients (86.4%) received HLA-mismatched and -related grafts, and 2 patients (4.5%) received HLA-matched and -unrelated grafts. The median age at allo-HSCT was 35 years (range, 16–67 years). The last follow-up was December 31, 2022 (Table 1). Patients who received HLA-mismatched and -unrelated grafts were treated with anti-thymocyte globulin. aGVHD prophylaxis regimens included cyclosporine A, short-term methotrexate, and mycophenolate mofetil. aGVHD staging and clinical responses to treatment were determined according to established guidelines (38).

To determine the effects of ruxolitinib on aGVHD BMSCs, 7 patients with aGVHD who were successfully treated with ruxolitinib as frontline therapy (detailed in Supplemental Table 1) and 5 patients with aGVHD who were followed up to 6 months after ruxolitinib treatment (detailed in Supplemental Table 2) were included in the analysis. We collected BM aspiration samples before and at least 2 weeks after ruxolitinib treatment (for short-term evaluation) and at least 6 months after ruxolitinib treatment (for long-term evaluation). Samples were immediately suspended in human BMSC culture medium consisting of DMEM/F-12 (Gibco, Thermo Fisher Scientific), 20% FBS (Gibco, Thermo Fisher Scientific), and 1% penicillin-streptomycin (Gibco, Thermo Fisher Scientific) and then filtered (0.2 mm pore size; BD Falcon). The medium was changed after 3 days of culturing, and BMSCs were harvested for further experiments, including colony formation, differentiation, and metabolism assays. Institutional databases were reviewed retrospectively to extract the demographic, clinical, and genetic data.

### Mice.

C57BL/6J (CD45.2^+^), BALB/C (CD45.2^+^), and B6.SJL-Ptprc^a^Pepc^b^/BoyJ (B6.SJL, CD45.1^+^) mice were purchased from the animal facility of the State Key Laboratory of Experimental Hematology (Tianjin, China). Gt (ROSA) 26Sortm1 (CAG-COX8A/Dendra2) Dcc (PhaM excised) mice containing the mitochondrial targeting signal of subunit 8a of cytochrome C oxidase (mito-Dendra2) were obtained from The Jackson Laboratory (stock no. 018397). Recipient CB6F1 (CD45.2^+^) mice were first-generation animals obtained by crossing male C57BL/6J mice with female BALB/C mice. Col2.3-GFP–transgenic mice were generally maintained on a CB6F1 background. All mice used in this study were 6–8 weeks old and maintained in a specific pathogen–free animal facility.

### BMT and establishment of the aGVHD model.

BMT and establishment of the aGVHD model were performed as previously described (7). Briefly, 4 hours after sublethal irradiation (4.0 Gy, twice), 5 × 10^6^ BM nucleated cells from female B6.SJL mice (CD45.1^+^) were intravenously injected into female CB6F1 recipients (CD45.2^+^) to establish the BMT model. Additional splenocytes (6 × 10^7^) from female C57BL/6 mice were injected into the BMT mice to establish the aGVHD model.

### Isolation of mouse niche cells.

Mice were sacrificed by CO_2_ asphyxia, and the unfractionated BM was crushed in digestion solution (3 mg/mL collagenase I, 3 mg/mL collagenase IV, and 1 mg/mL DNase I mixed in HBSS) and gently mixed by shaking at 37°C for 30 minutes to release BMSCs. To isolate OLCs, the bone matrix was chopped into pieces in another digestion solution (3 mg/mL collagenase I, 1 mg/mL DNase I, dispase II, mixed in HBSS) and gently mixed by shaking at 37°C for 30 minutes.

### scRNA-Seq.

Niche cells were enriched using a Lineage Cell Depletion Kit (Miltenyi Biotec) and isolated by FACS after labeling with antibodies for the detection of CD45 (30-F11), lineage mix (CD3e [17A], CD4 [GK1.5], CD8 [53-6.7], CD45R [RA3-6B2], Mac-1 [M1/70], Gr-1 [RB6-8C5], Ter-119 [TER-119]) and CD71 (RI7217) (all antibodies were obtained from BioLegend). Calcein AM and DAPI (MilliporeSigma) were used to identify and eliminate dead cells. Samples with a cell viability of greater than 80%, a cell concentration ranging from 700 cells/μL to 1,200 cells/μL, and cell diameters ranging from 5 μm to 40 μm were collected and loaded for sequencing. The cell suspension and gel beads contained barcode-sequencing–generated, single-cell gel bead-in emulsions for reverse transcription, and library preparation was performed using a Chromium Single Cell 30, version 2, Reagent Kit (10X Genomics). Sequencing was performed on an Illumina HiSeq PE150 or NovaSeq PE150 platform with 50,000–100,000 reads per cell and a 70%–80% saturation level, as recommended by 10X Genomics.

### Bioinformatics analysis of single-cell–sequenced reads.

Sequenced reads were trimmed using Trimmomatic software (Anthony M. Bolger, Trimmomatic) to produce clean data. For dimensionality reduction, cell filtering and *t*-distributed stochastic neighbor–embedding/ uniform manifold approximation and projection (*t*-SNE/UMAP) analysis followed by principal component analysis (PCA) were performed using Cell Ranger (10X Genomics). Graph-based clustering of the PCA-reduced data modified with the Louvain algorithm was visualized on a 2D *t*-SNE map (39). Nonhematopoietic cells were clustered according to reported transcriptomic signatures (14, 40). The same procedure was performed for subclustering. Force-directed layout embedding was based on ForceAtlas2 from the Gephi package. Pearson’s correlation analysis was applied on the basis of the average gene expression profile of each pair of clusters. Diffusion trajectory analysis was conducted according to a previously described method (41).

### Flow cytometry.

BM hematopoiesis was analyzed using the following antibodies (all from BioLegend): CD3e (clone 17A3), CD4 (clone GK1.5), CD8 (clone 53-6.7), CD45R (clone RA3-6B2), Mac-1 (clone M1/70), Gr-1 (clone RB6-8C5), Ter-119 (clone TER-119), Sca-1 (clone D7), CD117 (clone 2B8), CD34 (clone RAM34), CD150 (clone TC15-12F12.2), CD48 (clone HM48-1), CD41 (clone MWReg30), CD16/32 (clone 93), Flk2 (clone A2F10), IL-7R (clone A7R34), CD45.1 (clone A20), and CD45.2 (clone 104). BM niche cells were analyzed using the following antibodies (all from BioLegend, unless otherwise indicated): biotin-conjugated leptin receptor (R&D, catalog BAF497), CD31 (clone 390), CD51 (clone RMV-7), CD45 (clone 30-F11), and Ter119. Streptavidin-APC was used as an indirect labeling reagent for biotinylated antibodies. Dead cells were excluded by DAPI labeling (MilliporeSigma).

### Fibroblast CFU assay.

In total, 1,000,000 unfractionated BM niche cells from female CB6F1 mice were added to BMSC culture medium containing high-glucose DMEM, 20% FBS (Gibco, Thermo Fisher Scientific), and 1% penicillin-streptomycin (Gibco, Thermo Fisher Scientific) and then filtered (0.2 mm pore size; BD Falcon) in each well of a 6-well plate. After 14 days, the wells were washed with PBS, and cells were stained with crystal violet solution (MilliporeSigma) for 5 minutes at room temperature. Colonies containing more than 40 spindle-shaped cells were counted. For patient samples, 200,000 cells from the BM aspirates were plated immediately, and colonies were counted 14 days after seeding. The ratio of the number of colonies to the initial volume of the BM was used to assess the fibroblast CFU (CFU-F) ability.

### In vitro differentiation assay.

Unfractionated BM stromal cells were plated in a 10 cm^2^ dish containing BMSC culture medium (described in the CFU-F assay method). At 60%–70% confluence, cells were digested with 0.25% trypsin-EDTA and replated in collagen-coated 6-well plates. Osteogenic and adipogenic differentiation was induced using osteogenesis and adipogenesis differentiation kits (CYAGEN) according to the manufacturer’s instructions. Calcium nodule staining was performed with the von Kóssa method and Alizarin Red S for 20 minutes at room temperature. Fatty droplets were stained with Oil Red O for 20 minutes at room temperature.

### Bone sectioning and imaging.

Mouse femurs and tibiae were cleaned of muscle, fixed in 4% paraformaldehyde (MilliporeSigma) for 8 hours, and dehydrated in 30% sucrose solution for 24 hours. Bones were sectioned on a Leica CM1950 microtome and incubated in staining buffer (20% DMSO [MilliporeSigma], 5% donkey serum [Jackson ImmunoResearch], and 0.5% IGEPAL [MilliporeSigma]) in PBS containing primary antibodies for 8 hours. After washing with PBS and gentle shaking for 4 hours, the sections were incubated with secondary antibodies for 8 hours in the staining buffer mentioned above. After washing with PBS and gentle shaking for a further 4 hours, a rabbit anti-perilipin (MilliporeSigma, 1:400) primary antibody was used to label adipocytes, which were detected with a donkey anti–rabbit IgG (H+L) Alexa Fluor 555 (Thermo Fisher Scientific, 1:400) secondary antibody. DAPI was used to label all nucleated cells. Images were obtained using 2-photon excitation microscope (Olympus). 3D reconstruction was created using Velocity software (PerkinElmer).

### Calcein labeling.

On day 7 after modeling, calcein (10 mg/kg, in PBS) was subcutaneously injected into BMT and aGVHD mice. The mice were sacrificed on day 14 by CO_2_ asphyxia, and the femurs were isolated, fixed, sectioned, and stained with DAPI as described above. The distances between the calcein^+^ lines represented the rate of bone formation.

### Drug administration.

For PPARγ agonist treatment, rosiglitazone and GW1929 were dissolved in double-distilled H_2_O (ddH_2_O) and administered orally once a day from day 0 to day 20. Ruxolitinib was administered orally (dissolved in 30% PEG300, 2% DMSO, and ddH_2_O) twice per day from day 0 to day 20. Control aGVHD mice were administered vehicle (30% PEG300, 2% DMSO in ddH_2_O) on the same days. The body weight was recorded every day during the administration period.

### Intramedullary injection of BMSCs.

For intramedullary injection, BMSCs isolated from nontreated wild-type C57 mice were plated in BMSC culture medium containing vehicle (DMSO) or 0.5 μM ruxolitinib and then cultured and suspended at 1,000,000 cells per 5 μL in injection buffer (10 μM Y-27632 in HBSS; MilliporeSigma). For primary BMSC injection, cells were stained for biotin-conjugated leptin receptor (R&D Systems, BAF497), CD45 (BioLegend, 30-F11), and Ter119 (BioLegend, TER-119). Streptavidin-APC (BioLegend) was used as an indirect labeling reagent for biotinylated antibodies. Dead cells were excluded by labeling with DAPI (MilliporeSigma). CD45^–^Ter119^–^LepR^+^ cells were sorted and suspended at 10,000 cells per 5 μL in the same buffer. Then, BMSCs were injected intratibially into aGVHD mice at different time points using a micro-sample syringe (25 μL; Sangon Biotech).

### BMSC bulk RNA-Seq.

Total RNA was extracted from sorted or cultured BMSCs using a RNeasy Micro Kit (QIAGEN). Sequencing libraries were generated using a NEBNext Ultra RNA Library Prep Kit for Illumina (New England BioLabs [NEB]) following the manufacturer’s recommendations. After quantification and quality assessment, mRNA was purified with oligo-dT magnetic beads for library preparation. A total of 1 μg RNA per sample was used as input material for the RNA sample preparations. Divalent cations were used for fragmentation in NEBNext First Strand Synthesis Reaction Buffer (5×). First-strand cDNA was synthesized using a random hexamer primer and M-MuLV Reverse Transcriptase (RNase H, NEB). Second-strand cDNA synthesis was subsequently performed using DNA Polymerase I and RNase H. After the synthesis of both strands and adenylation of the 3′ ends of the DNA fragments, the NEBNext Adaptor with a hairpin loop structure was ligated to prepare the samples for hybridization. The library fragments were purified using an AMPure XP system (Beckman Coulter), and then 3 μL USER Enzyme (NEB) was mixed with the size-selected and adaptor-ligated cDNA (250–300 bp in length) at 37°C for 15 minutes, and then at 95°C for 5 minutes for PCR preparation. Next, PCR amplification was performed using Phusion High-Fidelity DNA polymerase, universal PCR primers, and an index (X) primer. The PCR products were purified using the AMPure XP system, and the library was assessed for further clustering and sequencing using an Agilent Bioanalyzer 2100 system. The index-coded samples were clustered using a TruSeq PE Cluster Kit v3-cBot-HS (Illumina), and library preparations were sequenced on an Illumina NovaSeq platform. Differential expression analysis was performed using the DESeq2 R package (version 1.16.1). Genes with an adjusted *P* value of less than 0.05, found by DESeq2, were assigned as differentially expressed and presented in a gene volcano map.

### RNA isolation and qPCR.

RNA was extracted from sorted BMSCs using a RNeasy Mini Kit (QIAGEN) and then reverse transcribed into cDNA with the Reverse Transcription System (Roche). qPCR was conducted using SYBR Green PCR Master Mix (ROCHE). The housekeeping gene *GAPDH* served as a positive quantitative control, and the relative quantitation of raw data was based on the ΔΔCt method. The primers used in this study were as follows: murine *Gapdh*, (forward) 5′-AAGTTCATCTGCACCACCG-3′ and (reverse) 5′-TGCTCAGGTAGTGGTTGTCG-3′; murine *Ppar*γ, (forward) 5′-ACCACTCGCATTCCTTTGAC-3′ and (reverse) 5′-TGGGTCAGCTCTTGTGAATG-3′; murine *Adipoq*, (forward) 5′-TGTTCCTCTTAATCCTGCCCA-3′ and (reverse) 5′-CCAACCTGCACAAGTTCCCTT-3′; murine *Runx2*, (forward) 5′-TTACCTACACCCCGCCAGTC-3′ and (reverse) 5′-TGCTGGTCTGGAAGGGTCC-3′; murine *Col2a1*, (forward) 5′-GTGGAGCAGCAAGAGCAAGGA-3′ and (reverse) 5′-CTTGCCCCACTTACCAGTGTG-3′; murine *Jak2*, (forward) 5’-CTCTCTGTCACAACCTCTTCGC-3′ and (reverse) 5′-TTGGTAAAGTAGAACCTCATGCG-3′; and human *ACTB*, (forward) 5′-AGCGAGCATCCCCCAAAGTT-3′ and (reverse) 5′-GGGCACGAAGGCTCATCATT-3′; human *ALP*, (forward) 5′-CAGAAGAAGGACAAACTGGG-3′ and (reverse) 5′-TTGTATGTCTTGGACAGAGC-3′; human *PPARG*, (forward) 5′-GAGCCCAAGTTTGAGTTTGC-3′ and (reverse) 5′-GCAGGTGTCTTGAATGTCTTC-3′; human *JAK2*, (forward) 5′-ATCCACCCAACCATGTCTTCC-3′ and (reverse) 5′-ATTCCATGCCGATAGGCTCTG-3′.

### Western blotting.

Western blotting was performed as described previously (42). In brief, total proteins were extracted from cells and resuspended in 5× SDS-PAGE loading buffer. The boiled protein samples were then subjected to SDS-PAGE followed by immunoblotting with the appropriate primary and secondary antibodies.

### Jak2-knockdown BMSC construction.

For lentivirus production, the pU6-MCS-mcherry-IRES-puromycin vector containing Jak2 shRNA or control lentivirus shRNA was transfected together with pSPAX2 and pMD2G into HEK293T cell lines using Lipofectamine 2000 (Thermo Fisher Scientific). Culture supernatants were harvested after 48 hours and 72 hours and concentrated using an Amicon filter (100K NMWL, MilliporeSigma). aGVHD BMSCs (third passage) were subsequently infected with lentiviral shRNA targeting Jak2 or the scrambled control and cultured in 10% FBS in DMEM containing 50 U/mL penicillin and 50 mg/mL streptomycin for 8 hours. The medium containing lentivirus was replaced with fresh medium, and the cells were cultured for a further 24 hours. Infected cells were then selected using puromycin (5 μg/mL) and cultured for 48 hours. The stable cell lines were used in functional assays.

### Mitochondrial ROS and membrane potential analyses.

Mitochondrial ROS and membrane potential were measured by MitoSox Red (Molecular Probes) and tetramethylrhodamine ethyl ester (TMRE) (Molecular Probes) staining, respectively. Following immunostaining for surface markers (CD45/Ter119/LepR and CD45.1/lineage/Sca-1/c-Kit), single-cell suspensions of BM or cocultured cells were incubated with MitoSox Red or TMRE in HBSS at 37°C for 30 minutes. Cells were washed twice with HBSS, labeled with DAPI, and analyzed using a BD FACSAria III (BD Biosciences). The MFI data were collated. Unlabeled cells were used as negative controls.

### Statistics.

Flow cytometry standard (FCS) files were analyzed using FlowJo software. Data are represented as the mean ± SEM. At least 3 independent replicates were included for all functional experiments. One-way ANOVA followed by an unpaired, 2-tailed *t* test was used to test for statistical differences. Multiple-testing correction was performed by the Holm-Bonferroni method. Survival was analyzed with the log-rank test. For scRNA-Seq data, the significance of gene expression between 2 group was compared using the Wilcoxon rank-sum test. In the calculation of the cumulative incidence of NRM, death and relapse were included as competing events, and survival was counted as a censored event. The NRM was compared between groups using Gray’s method. HRs were assigned 95% CIs (95% CI). A *P* value of less than 0.05 was considered statistically significant. All calculations were performed using SPSS version 20 (IBM) and R version 3.6.1 (R Foundation for Statistical Computing) software programs.

### Study approval.

All human studies were approved by the IRBs of Shanghai Rui Jin Hospital (Shanghai, China) and the Institute of Hematology and Blood Diseases Hospital, the Chinese Academy of Medical Sciences, and Peking Union Medical College (Tianjin, China). Each patient (or a legal guardian) provided signed informed consent for therapy, and the collection of prospective data was conducted in accordance with the Declaration of Helsinki. Animal experiments were approved by the IACUC of the State Key Laboratory of Experimental Hematology and the Institute of Hematology (Tianjin, China).

### Data availability.

The scRNA-Seq (10XGenomics) and bulk RNA-Seq data can be accessed in the NCBI’s Gene Expression Omnibus (GEO) database (GEO GSE157389, GSE157326, and GSE165413). The original data can be accessed in the Supporting Data Values file.

## Author contributions

Y Lin, QG, SHL, ZKP, and ZNY designed the study, performed the experiments, analyzed the data, and wrote the manuscript. YPL, SDY, ZFZ, GHS, FLG, CX, XNZ, FJW, and SRY, helped with the mouse experiments. YLL and CCW helped with single-cell analysis. Y Liu, YC, XBX helped with patient sample experiments. WYG, TC, HC, and XXH proposed and designed the study, interpreted the results, wrote the manuscript, and oversaw the project. The order of the co–first authors’ names was determined on the basis of their relative contributions to the study.

## Supplementary Material

Supplemental data

Supporting data values

## Figures and Tables

**Table 1:**
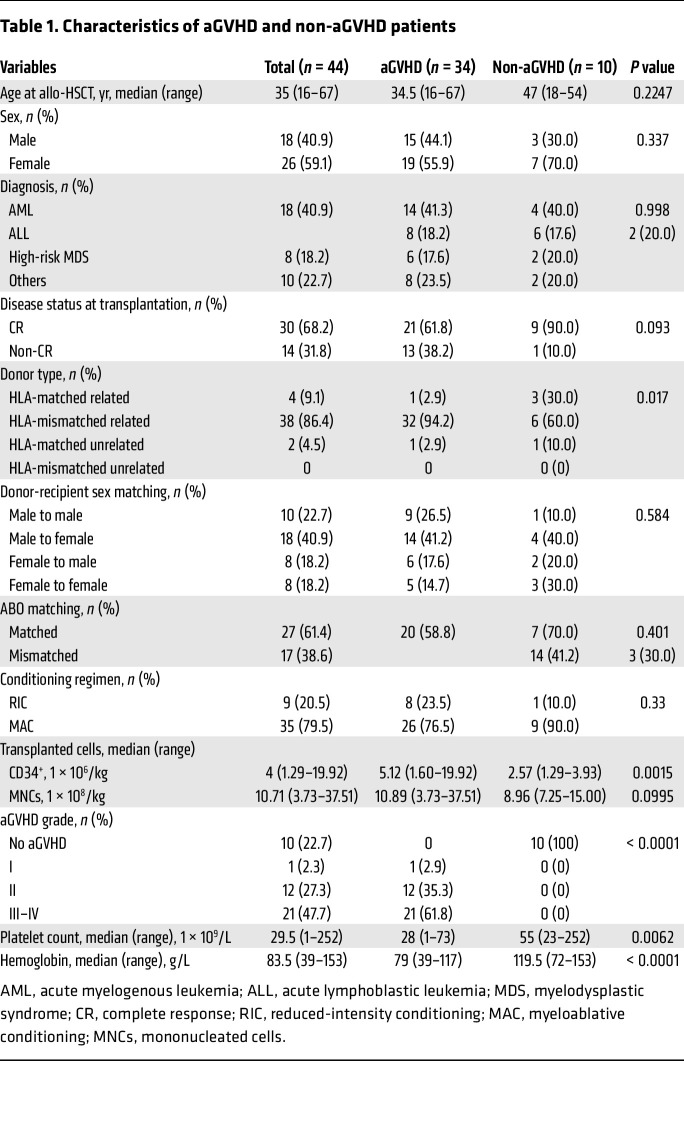
Characteristics of aGVHD and non-aGVHD patients

## Version 1 06/20/2023

In-Press Preview

## Version 2 08/01/2023

Electronic publication

## Version 3 11/17/2025

Correction to Figure 9B

## Version 4 08/17/2026

Corrected errors in the raw data in Figure 5C and sdvals
