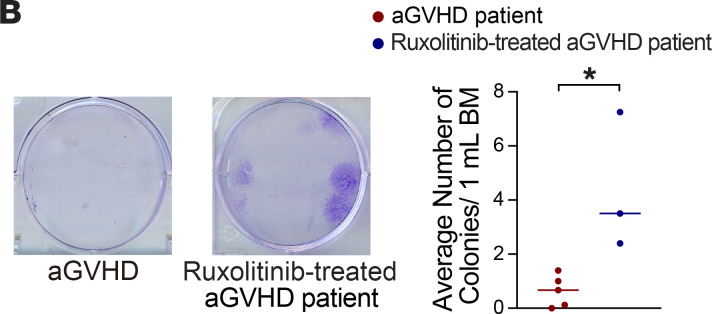# Corrigendum to Ruxolitinib improves hematopoietic regeneration by restoring mesenchymal stromal cell function in acute graft-versus-host disease

**DOI:** 10.1172/JCI200847

**Published:** 2025-11-17

**Authors:** Yan Lin, Quan Gu, Shihong Lu, Zengkai Pan, Zining Yang, Yapu Li, Shangda Yang, Yanling Lv, Zhaofeng Zheng, Guohuan Sun, Fanglin Gou, Chang Xu, Xiangnan Zhao, Fengjiao Wang, Chenchen Wang, Shiru Yuan, Xiaobao Xie, Yang Cao, Yue Liu, Weiying Gu, Tao Cheng, Hui Cheng, Xiaoxia Hu

Original citation: *J Clin Invest*. 2023;133(15):e162201. https://doi.org/10.1172/JCI162201

Citation for this corrigendum: *J Clin Invest*. 2025;135(22):e200847. https://doi.org/10.1172/JCI200847

The authors recently became aware that the aGVHD image in Figure 9B was inadvertently duplicated from the aGVHD + vehicle image in 9D. The correct (Figure 9B) provided from the original source data is below. The HTML and PDF versions of the paper have been updated.

The authors regret the error.

## Figures and Tables

**Figure 9B:**