# Second corrigendum to Ruxolitinib improves hematopoietic regeneration by restoring mesenchymal stromal cell function in acute graft-versus-host disease

**DOI:** 10.1172/JCI210543

**Published:** 2026-08-17

**Authors:** Yan Lin, Quan Gu, Shihong Lu, Zengkai Pan, Zining Yang, Yapu Li, Shangda Yang, Yanling Lv, Zhaofeng Zheng, Guohuan Sun, Fanglin Gou, Chang Xu, Xiangnan Zhao, Fengjiao Wang, Chenchen Wang, Shiru Yuan, Xiaobao Xie, Yang Cao, Yue Liu, Weiying Gu, Tao Cheng, Hui Cheng, Xiaoxia Hu

Original citation: *J Clin Invest.* 2023;133(15):e162201. https://doi.org/10.1172/JCI162201

Citation for this corrigendum: *J Clin Invest.* 2026;136(16):e210543. https://doi.org/10.1172/JCI210543

The authors recently identified errors in the raw data underlying Figure 5C that occurred due to a copy-and-paste error made during data processing. The correct Figure 5C is shown below. The HTML and PDF versions of the paper as well as the accompanying Supporting Data Values file have been updated. Note that a prior correction for this paper was published in 2025 (1).

The authors regret the error.

## Supplementary Material

Supporting data values

## Figures and Tables

**Figure 5C:**
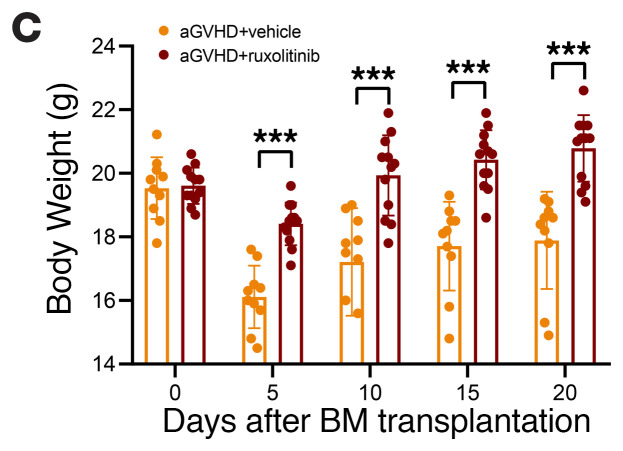


## Version 1 08/17/2026

Electronic publication
