# Supplementary material for: Ruxolitinib improves hematopoietic regeneration by restoring mesenchymal stromal cell function in acute graft-versus-host disease
Source: J Clin Invest. 2023 Jun 20;133(15):e162201. doi: 10.1172/JCI162201 (PMC10378163; doi:10.1172/JCI162201)
Supplement: Supplemental data [file jci-133-162201-s011.pdf]

Supplemental information

Supplemental Figure 1. Transcriptional remodeling of the BM niche in aGVHD mice.

Supplemental Figure 2. Alterations to cellular components in the aGVHD BM niche.

Supplemental Figure 3. PPARγ agonists were not sufficient to prolong the survival of aGVHD mice.

Supplemental Figure 4. Protective effect of ruxolitinib on hematopoiesis and the BM niche in aGVHD.

Supplemental Figure 5. BMSC injection.

Supplemental Figure 6. Ruxolitinib inhibited activation of the JAK2/STAT1 pathway in aGVHD BMSCs.

Supplemental Figure 7. Ruxolitinib enhanced aGVHD patient-derived BMSC function in the long-term follow-up.

Supplemental Table 1. Characteristics of aGVHD patients with ruxolitinib treatment.

Supplemental Table 2. Clinical and biological characteristics of long-term follow-up aGVHD patients after ruxolitinib treatment.

Supplemental Figure 1. Transcriptional remodeling of the BM niche in aGVHD mice.

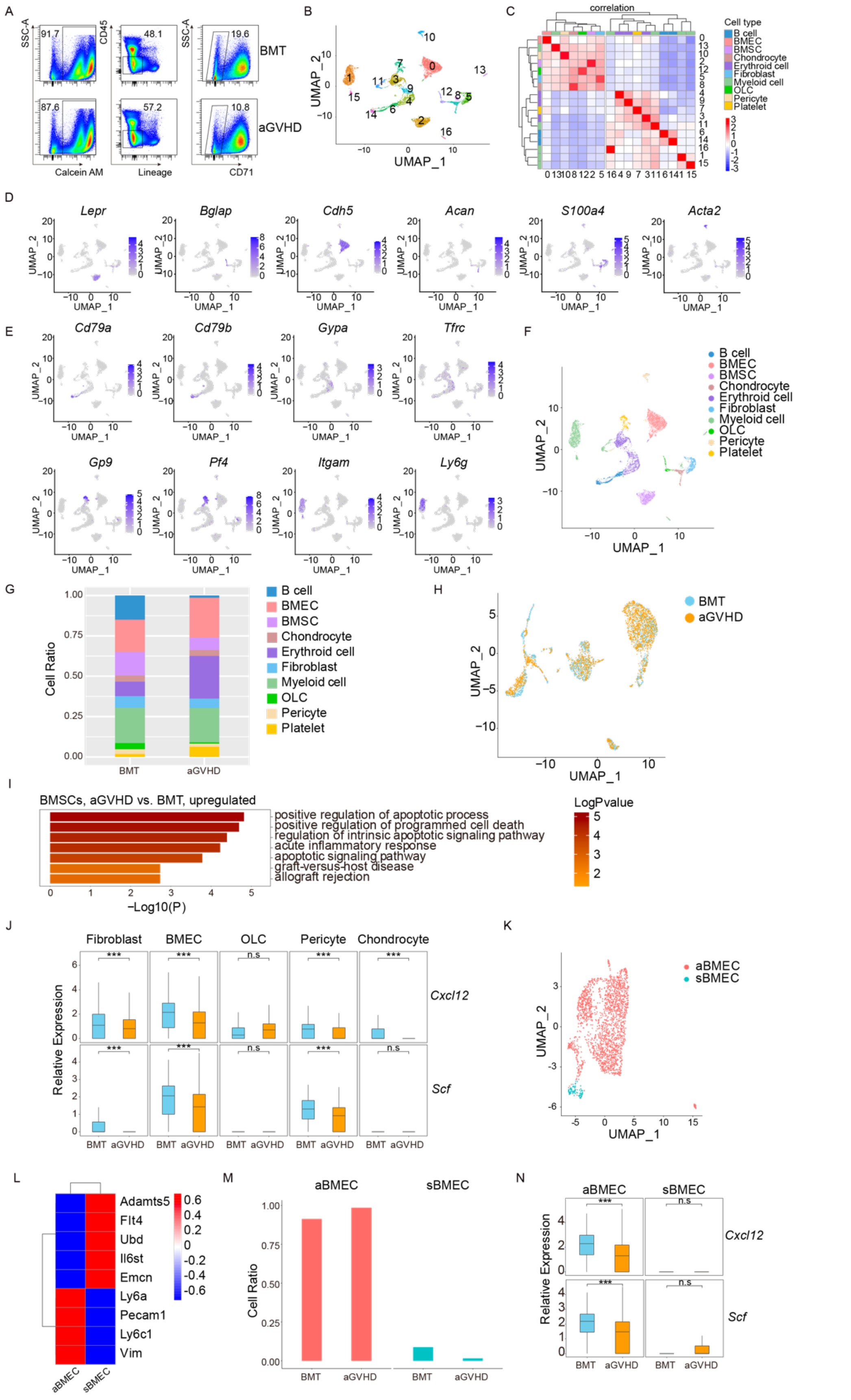

(A) FACS sorting strategy of niche cells from the BMT and aGVHD groups. BMT, n = 10; aGVHD, n = 16. **(B)** t-SNE map of all cells isolated from BMT and aGVHD mice. **(C)** Pearson's correlation between clusters (based on the average gene expression profiles). **(D** and **E)** Specifically expressed markers of niche cells **(D)** and hematopoietic cells **(E)**. **(F)** t-SNE map of all sorted cells; different cell types are colored-coded. **(G)** Cellularity in each cluster. **(H)** t-SNE map of niche cellular components in BMT and aGVHD; different samples are colored-coded. **(I)** GO term enrichment of significantly upregulated genes in aGVHD BMSCs. **(J)** Alterations to niche factor (*Cxcl12* and *Scf*) expression in niche cells. **(K)** Sub-clustering of BMECs. aBMEC, arteriolar BMEC, sBMEC, sinusoidal BMEC. **(L-N)** Gene signature **(L)**, cell ratio **(M)** and niche factor expression **(N)** of BMEC sub-populations from BMT and aGVHD mice. $^{**}P < 0.01$, $^{***}P < 0.001$, n.s, not significant. Wilcoxon Rank-Sum test was used in **(J** and **N)**.

Supplemental Figure 2. Alterations to cellular components in aGVHD BM niche.

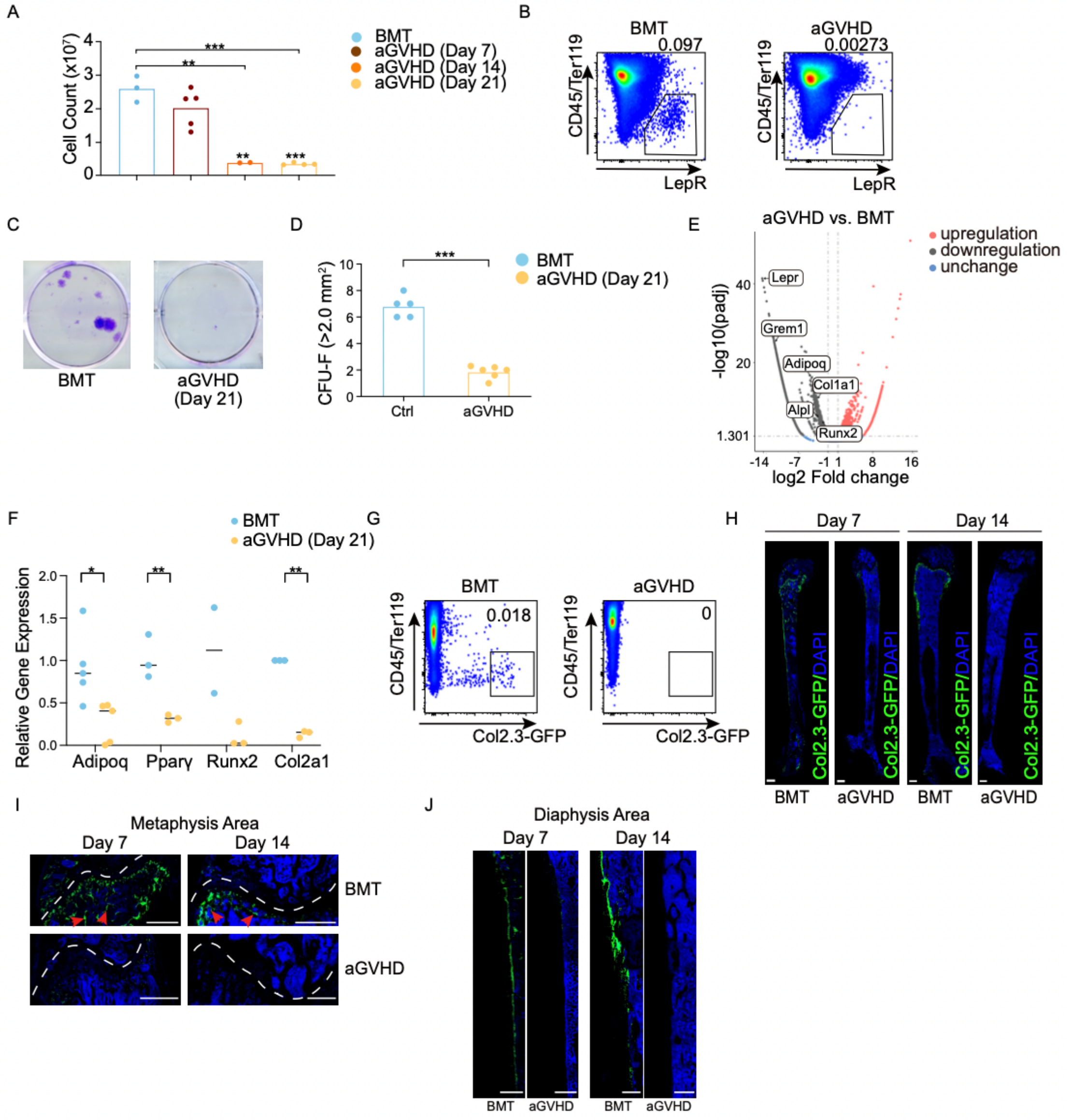

(A) Absolute number of niche cells at different time points; n = 10–15 per group. **(B)** Representative flow cytometry plots of LepR^{+} cells. Cells were pre-gated on singlet live cells. Three independent replicates. **(C)** Representative image of BMSC colony-forming assay. (D) Quantitative assay of CFU-F. **(E)** Volcano map of differentially expressed genes between BMT and aGVHD BMSCs. **(F)** mRNA expression of BMSC differentiation-related genes; n = 20 per group. **(G)** Representative flow cytometry plots of Col2.3-GFP^{high} cells. Three independent replicates. (H) Immunofluorescent images of femurs at day 7 and day 14; three

independent replicates. Scale bar, 500 μm. **(I** and **J)** High-magnification views of metaphysis areas **(I)** and diaphysis areas **(J)**. White dotted lines represent growth plate and red arrows indicate Col2.3-GFP^{high} osteoblasts. Scale bar, 200 μm. *$P < 0.05$, **$P < 0.01$, ***$P < 0.001$. 1-way ANOVA followed by unpaired t test was used in **A**. Unpaired t test was performed in **(D** and **F)**.

Supplemental Figure 3. PPARγ agonists were not sufficient to prolong the survival of aGVHD mice.

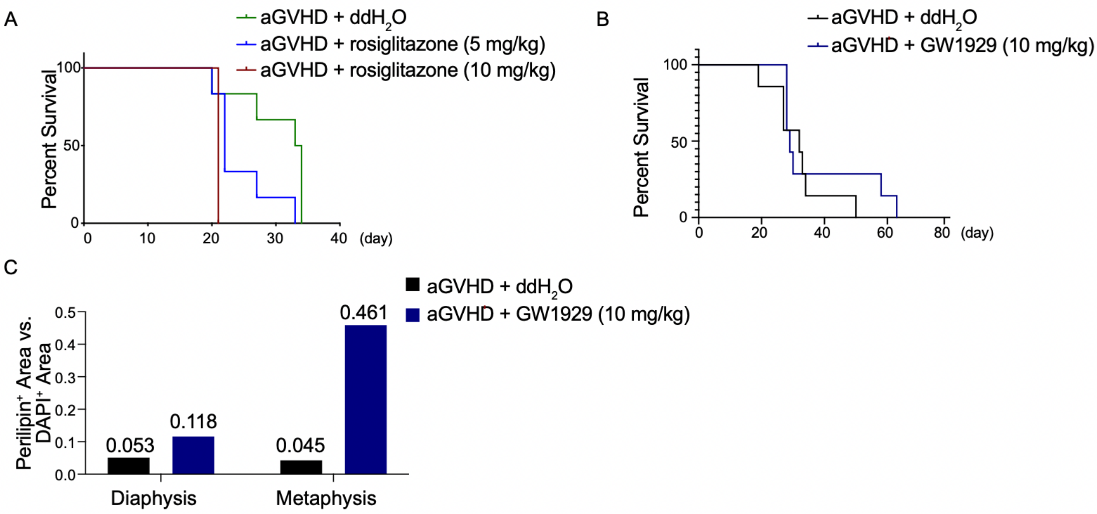

(A and **B)** Survival curve of aGVHD mice after rosiglitazone **(A)** and GW1929 **(B)** administration; n = 7–13 per group. **(C)** Quantitative analysis of perilipin$^+$ area vs. DAPI$^+$ area after GW1929 treatment. Log-rank test was used in (**A** and **B**).

Supplemental Figure 4. Protective effect of ruxolitinib on hematopoiesis and the BM niche in aGVHD.

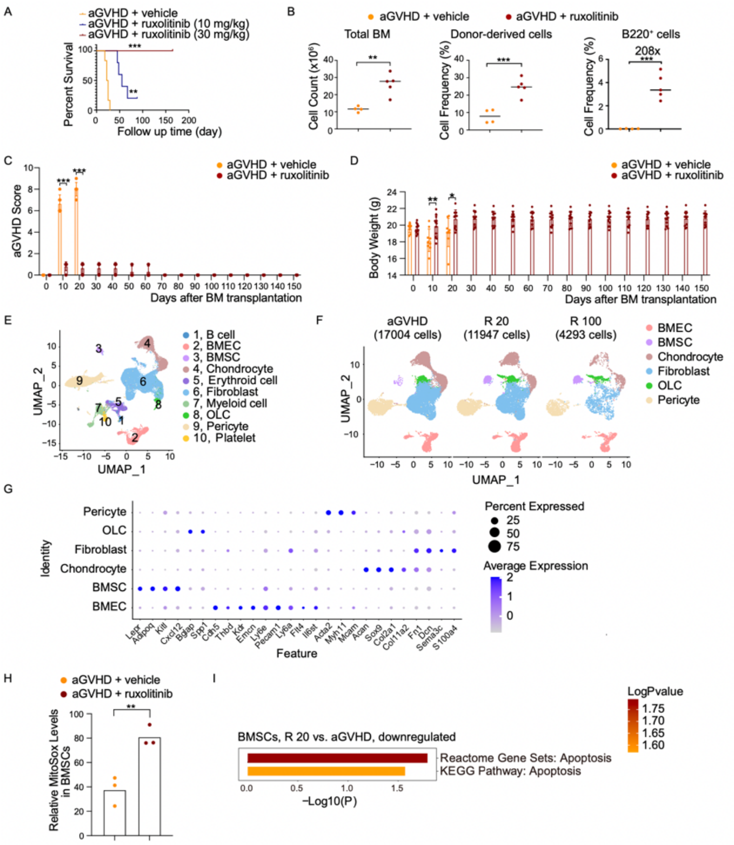

(A) Survival curve of aGVHD mice after vehicle or ruxolitinib treatment (10 mg/kg or 30 mg/kg, oral administration, twice a day); n = 6 per group. **(B)** BM cellularity, donor-derived cells and B220$^+$ B cell frequency in vehicle- or ruxolitinib-treated aGVHD mice (30 mg/kg, oral administration, twice a day). aGVHD+vehicle, n = 4, aGVHD+ruxolitinib, n = 5. **(C and D)** aGVHD score **(C)** and bodyweight **(D)** in the long-term follow-up. n = 9 per group. **(E)** UMAP visualization of hematopoietic and stromal cells. **(F)** UMAP visualization of niche cells at different time points. **(G)** Cluster signature genes of niche cells. **(H)** Relative MitoSox level in BMSCs derived from vehicle- or ruxolitinib-treated aGVHD mice; n = 3. **(I)** Enrichment of downregulated genes in ruxolitinib-treated aGVHD BMSCs. R 20, Day 20 post-transplantation. **P* < 0.05, ***P* < 0.01, ****P* < 0.001. Log-rank test was used in **A**. Unpaired t test was performed in **(B**, **C** and **H)**.

Supplemental Figure 5. BMSC injection.

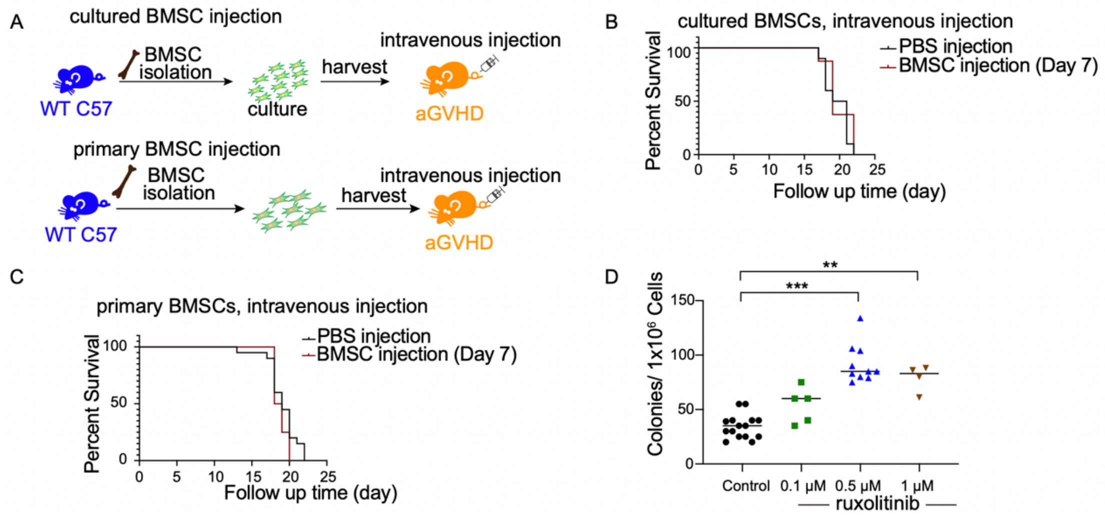

(A) Intravenous BMSC injection protocol. **(B** and **C)** Survival curve of aGVHD mice after intravenous injection of cultured BMSCs **(B)** and primary BMSCs **(C)**; n = 6–10 per group. **(D)** Colony forming ability of BMSCs after treatment with different doses of ruxolitinib before injection; n = 4–15 per group. ***P* < 0.01, ****P* < 0.001. Log-rank test was used in **(B** and **C)**. 1-way ANOVA followed by unpaired t test was used in **D**.

Supplemental Figure 6. Ruxolitinib inhibited the activation of JAK2/STAT1 pathway in aGVHD BMSCs.

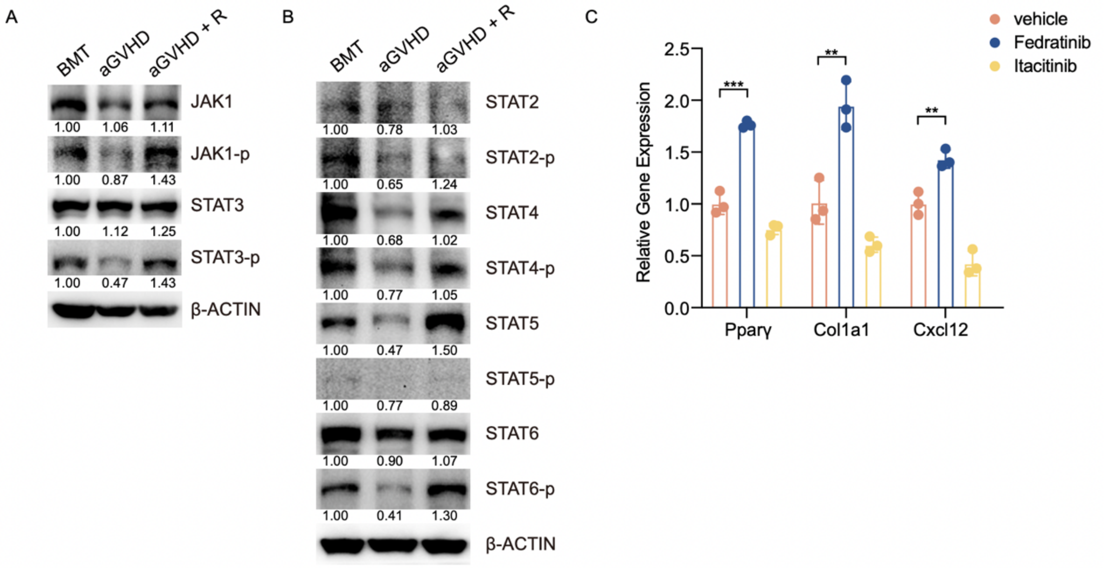

(A-B) Activation of JAK1/STAT3 (A) and other STAT family members (B) in BMSCs derived from BMT, aGVHD and ruxolitinib-treated aGVHD mice. BMT, n = 10, aGVHD, n = 15, aGVHD+R, ruxolitinib-treated aGVHD mice. n = 10. Numbers above columns represent the fold change compared to BMT mice. (C) Relative differentiation- and hematopoietic supportive-related gene expression of aGVHD BMSCs after in vitro treatment with vehicle, Fedratinib (0.5 μM) or Itacitinib (0.5 μM) for 24 h; n = 3 per group. Unpaired t test was performed in **C**.

Supplemental Figure 7. Ruxolitinib enhanced aGVHD patient-derived BMSC function in the long-term follow-up.

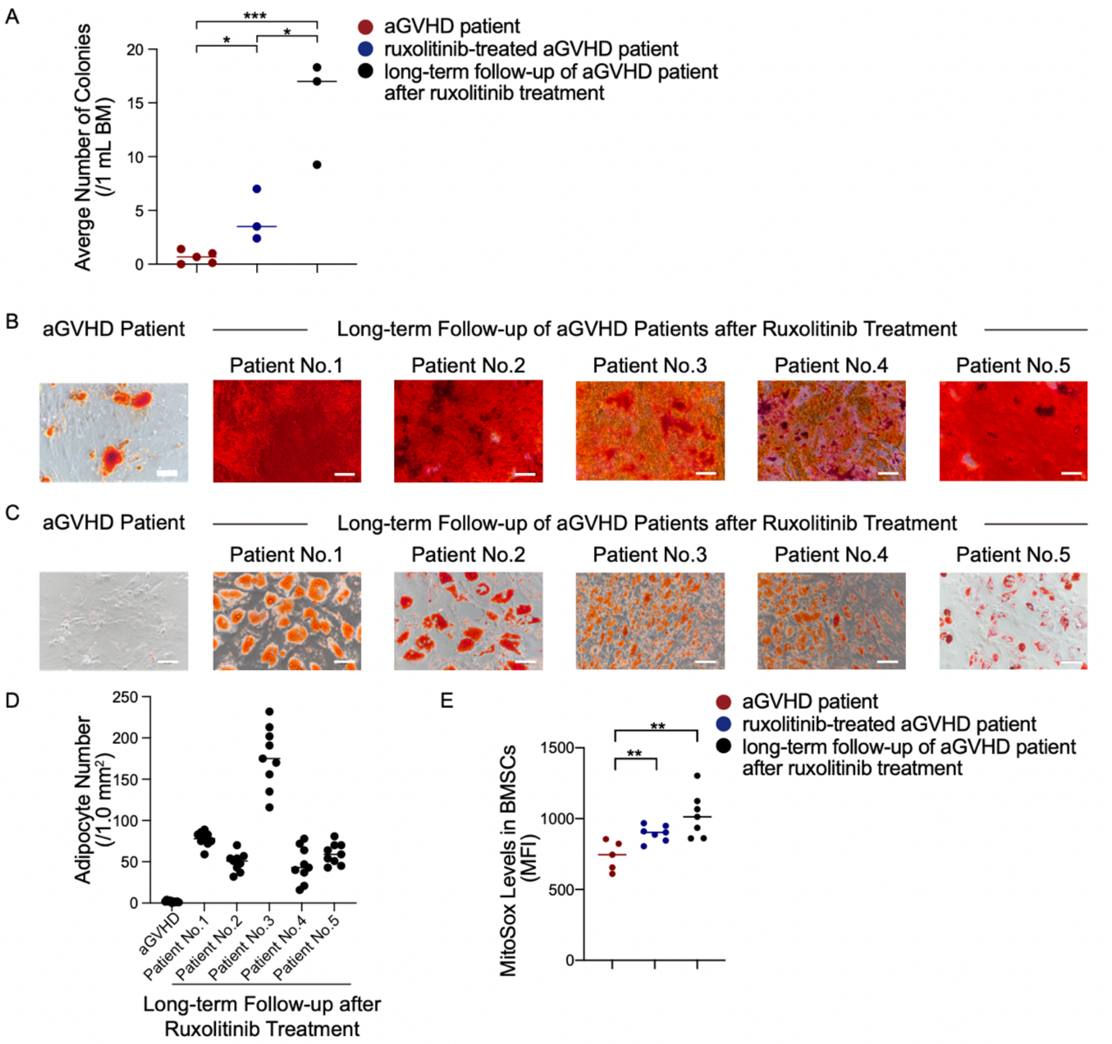

(A) Colony-forming ability of aGVHD patients and long-term follow-up ruxolitinib-treated aGVHD patients; n = 3–5 per group. **(B and C)** Representative images of the osteogenesis **(B)** and adipogenesis **(C)** ability of aGVHD patients and long-term follow-up ruxolitinib-treated aGVHD patients. Scale bar, 200 μm. Three independent replicates. **(D)** Quantitative assay of adipogenesis potential. **(E)** Mitochondrial ROS level in BMSCs; n = 5–7 per group. **P* < 0.05, ***P* < 0.01, ****P* < 0.001. 1-way ANOVA followed by unpaired t test was used in (**A** and **E**).

Supplemental Table 1. Characteristics of aGVHD patients with ruxolitinib treatment

Patient number	Sex	Age (years)	Diagnosis	Donor type	Graft type	Conditioning regimen	aGVHD prophylaxis	aGVHD onset time	aGVHD grade, organ	aGVHD treatment outcome
1	M	40	AML	HLA-matched, related	PBSC	MAC	ATG based, CsA+MPA+MTX	d15	Grade II (G1S0L0)	CR
2	M	25	AML	HLA-mismatched, related	PBSC	MAC	ATG based, CsA+MPA+MTX	d23	Grade II (G1S0L0)	CR
3	M	56	AML	HLA-mismatched, related	PBSC	MAC	ATG based, CsA+MPA+MTX	d20	Grade III (G2S1L0)	PR
4	M	56	MPAL	HLA-mismatched, related	PBSC	MAC	ATG based, CsA+MPA+MTX	d16	Grade IV (G4S2L0)	PR
5	F	55	AML	HLA-mismatched, related	PBSC	MAC	ATG based, CsA+MPA+MTX	d22	Grade III (G2S1L0)	CR
6	M	32	AML	HLA-mismatched, related	PBSC	MAC	ATG based, CsA+MPA+MTX	d24	Grade II (G0S3L0)	CR
7	F	42	T-LBL/T-ALL	HLA-mismatched, related	PBSC	MAC	ATG based, CsA+MPA+MTX	d19	Grade IV (G4S0L0)	CR

M, male; F, female; PBSC, peripheral blood stem cell; AML, acute myelogenous leukemia; MPAL, mixed phenotype acute leukemia; T-LBL/T-ALL, T cell lymphoblastic lymphoma/T cell acute lymphoblastic leukemia; HLA, human leukocyte antigen; MAC, myeloablative conditioning; ATG, anti-thymocyte globulin; CsA, cyclosporine; MPA, mycophenolic acid; MTX, methotrexate; G, gastrointestinal tract; S, skin; L, liver; CR, complete response; PR, partial response.

Supplemental Table 2. Clinical and biological characteristics of long-term follow-up aGVHD patients after ruxolitinib treatment

Patient number	Sex	Age (years)	Diagnosis	Donor type	Graft type	Conditioning regimen	aGVHD prophylaxis	aGVHD onset time	aGVHD grade, organ	aGVHD treatment outcome
1	M	21	AML	HLA-matched, unrelated	PBSC	MAC	ATG based, CsA+MPA+MTX	d13	Grade II (G0S3L0)	CR
2	F	39	T-LBL/ T-ALL	HLA-mismatched, related	PBSC	MAC	ATG based, CsA+MPA+MTX	d17	Grade III (G3S0L0)	CR
3	M	21	AML	HLA-mismatched, related	PBSC	MAC	ATG based, CsA+MPA+MTX	d14	Grade II (G1S0L0)	CR
4	M	56	AML	HLA-mismatched, related	PBSC	MAC	ATG based, CsA+MPA+MTX	d16	Grade IV (G4S2L0)	CR
5	M	40	AML	HLA-matched, related	PBSC	MAC	ATG based, CsA+MPA+MTX	d47	Grade III (G0S1L2)	CR

M, male; F, female; PBSC, peripheral blood stem cell; AML, acute myelogenous leukemia; T-LBL/T-ALL, T cell lymphoblastic lymphoma/T cell acute lymphoblastic leukemia; HLA, human leukocyte antigen; MAC, myeloablative conditioning; ATG, anti-thymocyte globulin; CsA, cyclosporine; MPA, mycophenolic acid; MTX, methotrexate; G, gastrointestinal tract; S, skin; L, liver; CR, complete response.
